# Patient Perspectives on Health Insurance Design: A Mixed-Methods Analysis

**DOI:** 10.3390/jmahp13040056

**Published:** 2025-11-14

**Authors:** Bridget Doherty, Kimberly Hooks, Ulrich Neumann, Wesley Peters, Steven Zona, Lisa Shea

**Affiliations:** 1 Johnson & Johnson, Titusville, NJ 08560, USA; uneuman1@its.jnj.com (U.N.); szona@its.jnj.com (S.Z.); lshea4@its.jnj.com (L.S.); 2 Patient Author, Powder Springs, GA 30127, USA; kimberlymhooks@gmail.com; 3 Evidera, Wilmington, NC 28401, USA; wesley.peters@evidera.com

**Keywords:** insurance reform, patient perspectives, healthcare policy, mixed-methods research, utilization management, cost sharing, out-of-pocket cost, patient engagement

## Abstract

There remains a persistent lack of patient-centered evidence on insurance reform and real-world experiences of patients with chronic disease. This study gathered insights around insurance design from chronic disease beneficiaries. This mixed-methods analysis comprised an online survey and virtual focus group sessions (August to December 2023) involving US residents with chronic disease and health insurance. Patients’ perspectives on insurance design were explored. Survey data were analyzed descriptively. Key themes were identified from focus group transcripts and direct observations. In total, 146 patients across 15 chronic diseases completed the survey; 29 then participated in focus groups. Although most beneficiaries were satisfied with their health plan, concerns centered on prescription medication affordability due to high deductibles and cost exposure, the disproportionate effects of cost exposure based on income, and the unpredictability of out-of-pocket costs. For some, the financial burden led to financial debt, therapy abandonment, mental health issues, and/or worsening of their condition. Overall, there was broad support for policy solutions to redesign insurance and adjust cost exposure for patients with chronic disease. This research offers valuable patient insights into health insurance design in the US to ensure patients’ needs are addressed.

## 1. Introduction

More than 90% of Americans have health insurance [1], but many struggle with coverage and affordability issues such as high out-of-pocket (OOP) costs, complex utilization management or prior authorization processes, and denial of prescribed medication. It is estimated that 45% of US adults aged 19–64 live in families facing financial difficulties related to healthcare [2], and nearly one-quarter (23%) of US adults face medical debt [3] even though many of them have insurance.

In health insurance, the concept of “moral hazard” refers to the idea that coverage without OOP costs to patients may result in excess healthcare utilization beyond economically efficient levels, stemming from either patient overuse or provider overservicing [4,5]. To manage moral hazard, US insurance design has evolved to incorporate increasing obligations of patient cost exposure, including copays, coinsurance, and deductibles—amounting to a historic total of US$471 billion in 2022 [6]. Another form of moral hazard has been proposed with respect to profit-maximizing health plans, which may disadvantage sick policyholders by denying or delaying legitimate coverage [7]. Together, these concepts have shaped policy discussions on which strategies are best for the optimization of efficient healthcare use and value-based care, with recent evidence suggesting that wide implementation of patient cost exposure is not well supported by empirical evidence when assessing the use of valuable, clinically appropriate services in the current US health insurance landscape [8]. Indeed, patients may broadly value the financial protection offered by generous coverage, even if they need to pay more in premiums [9], supporting a need for a more nuanced approach to cost sharing.

Importantly, policy discussions usually center around “average” patients, often without discussion of patient heterogeneity. However, certain populations, including people with multiple chronic diseases, are more vulnerable than others to the burden of OOP costs, healthcare affordability, and, consequently, the threat of medical debt [10,11,12,13]. It is estimated that 60% of Americans have a chronic illness [14]. Patient cost exposure in insurance, traditionally rationalized as a mechanism to reduce overuse of healthcare resources, disproportionately burdens beneficiaries who have chronic disease, leaving them with substantial, often unaffordable costs [15,16]. This phenomenon, sometimes termed “insured but not covered”, raises questions about how US health insurance designs address the needs of beneficiaries with chronic disease [16].

Recent evidence suggests that this approach may pose a barrier to affordable and accessible care, disproportionately burdening those with the fewest resources, the highest medical utilization, and the least potential for moral hazard-driven excess use [8]. Patients with chronic disease requiring continuous medical treatment are already subject to stringent utilization management prior to therapy use, and are a group disproportionately burdened by customary cost-sharing obligations. Additionally, patients are rarely given a voice in discussions that take place in the academic setting about optimizing insurance. There is an opportunity to reform health insurance by adopting a clinically driven, value-based design. Without patient feedback, policies may be enacted without reference to the insights and input of patients who are most affected.

This analysis gathered quantitative and qualitative insights around insurance design from chronic disease beneficiaries. The aim was to elucidate their experiences and perspectives around health insurance policies and aspects of moral hazard to help inform future insurance design and reform.

## 2. Materials and Methods

### 2.1. Study Design

**Figure 1:**
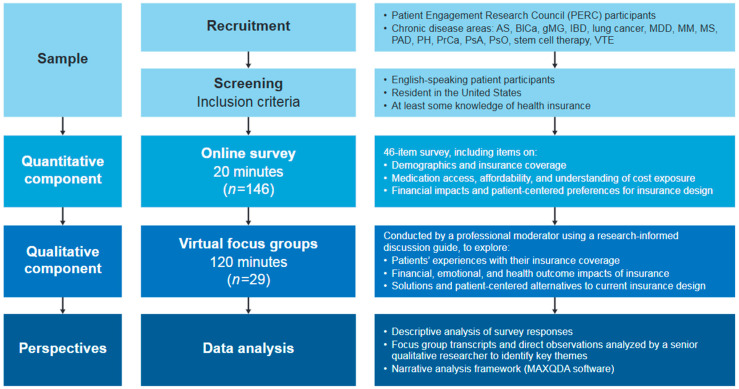
Research methodology. AS, ankylosing spondylitis; BICa, bladder cancer; gMG, generalized myasthenia gravis; IBD, inflammatory bowel disease; MDD, major depressive disorder; MM, multiple myeloma; MS, multiple sclerosis; PAD, peripheral artery disease; PH, pulmonary hypertension; PrCa, prostate cancer; PsA, psoriatic arthritis; PsO, psoriasis; VTE, venous thromboembolism.

A mixed-methods analysis was conducted in two parts (Figure 1). First, an initial online survey was designed to gain quantitative insights from a broad and diverse group of patients with chronic disease. Secondly, live, virtual focus group sessions were conducted with a smaller subset of patients to gather more in-depth qualitative insights around issues identified in the survey.

All patient data were de-identified, and the study was exempt from institutional review board review pursuant to the terms of the U.S. Department of Health and Human Services’ Policy for Protection of Human Research Subjects at 45 C.F.R. §46.104(d). The study was conducted in accordance with the Helsinki Declaration of 1964 and its later amendments. This study is reported according to:

1. The Guidance for Reporting Involvement of Patients and the Public (GRIPP2) [17];
2. The STrengthening the Reporting of OBservational studies in Epidemiology (STROBE) [18]; and
3. The COnsolidated criteria for REporting Qualitative research (COREQ) [19].

### 2.2. Study Participants

Patients with chronic disease residing in the United States and participating in Johnson & Johnson’s established Patient Engagement Research Council (PERC) program [20] were eligible to participate. Johnson & Johnson’s PERC includes a diverse group of disease-aware participants living with chronic health conditions who provide their insights and feedback around a specific, structured series of activities [21,22,23,24]. PERC members include both patients and caregivers with a range of demographics, disease awareness, and healthcare experiences, some more involved in healthcare decision-making than others. Recruitment targets “everyday” participants through outreach to social media, websites, and patient support groups. Interested participants undergo a screening and interview process before selection that includes discussion of any prior research study participation. PERC members provide written informed consent and are compensated for their time; participation in each research opportunity is voluntary. At the time of this study, the PERC program consisted of 218 participants.

### 2.3. Survey

All eligible PERC members were invited via email to complete a 46-item online survey (File S1) between 30 August and 6 September 2023. In addition to capturing information on demographics and insurance coverage, survey questions explored participants’ perspectives on medication access, affordability, understanding of cost exposure, financial impact (and consequences of this), and preferences on potential changes to current insurance design. Respondents who indicated having no knowledge of their insurance coverage were excluded from the survey after the first question. Caregivers were also excluded from the survey, to focus on the direct patient perspective.

### 2.4. Focus Group Sessions

After completion and analysis of the survey, six live, virtual focus group sessions were held with selected groups of the survey respondents between 29 November and 7 December 2023. To ensure diversity, participants were chosen based on demographics (age, race/ethnicity, gender, disease area) and their survey responses about insurance coverage. To prevent bias, research was designed with multiple data collection methods (survey and focus groups) and multiple reviewers analyzed the research. The focus groups were designed to:

1. Further explore patients’ experiences with their insurance coverage;
2. Understand the financial, emotional, and health outcome impacts of patients’ current insurance coverage; and
3. Explore solutions and patient-centered alternatives to current insurance design.

Six separate focus group sessions (each lasting 120 min) were scheduled to enable robust discussion among five or six patients at a time. At least one member of the sponsor’s scientific team attended each session. Three sessions included patients with commercial insurance and three included those on Medicare with or without Medicaid or secondary insurance. Sessions were conducted by a research specialist from Evidera (part of Thermo Fisher Scientific, LLC) with more than 8 years’ experience in qualitative, patient-centered healthcare research. The moderator independently directed the focus groups using a discussion guide developed by an expert in research methods for patients (File S2). At the beginning of the session, the researcher introduced himself and explained the goals of the research. The findings of the survey were discussed, and participant feedback was invited. The topics included costs for prescription medication, value of insurance coverage, access restrictions, impact of costs, impact to mental and physical health, the future of insurance, and potential solutions for insurance reform. Sessions were audio recorded and transcribed; field notes were consulted for preliminary impressions. Transcripts were not shared with the participants. After completion of the six focus group sessions, the team discussed and agreed that there was no need for additional sessions based on data saturation.

### 2.5. Data Analysis

Survey results were compiled by the research specialist and were analyzed descriptively. Data collection and analysis from the focus groups were led by a research specialist experienced in narrative analysis and drawing insights from interactions with patients, with support from a research associate. As previously described [25], data analysis involved applying a narrative analysis framework to review and code transcripts based on a priori topics and lived experiences of participants. This was a systematic approach involving post-implementation review qualitative analysis methodology. A deductive coding process was applied, using codes derived from the interview guide to identify common themes and findings across the research materials. Coding was conducted independently by two qualitative researchers to mitigate potential bias. Key themes were identified using qualitative research analysis software (MAXQDA, Version 24; VERBI Software, Berlin, Germany). The research specialists analyzed the themes derived from transcript coding and sponsor review to synthesize findings in a written report.

While there has been past criticism surrounding the use of the terms “OOP costs” and “cost sharing” [26], the terminology used in this analysis is typically encountered by patients in insurance design documents. “Cost sharing” was used as the umbrella term for both fixed copays and percentage-based coinsurance rates, except for those questions where their impact was specifically evaluated; in these cases, the individual terms were noted. Similarly, affordability in healthcare is a subjective concept that is difficult to define, widely considered to be a function of income, spending, and perception of value [27]. In this analysis, the term “affordable” was used broadly as an economic concept representing the ability to pay both annual premiums and OOP expenses without reducing spending on basic necessities, such as childcare, food, housing, and transportation.

## 3. Results

### 3.1. Patients

In total, 204 patient members of the PERC program (*N* = 218) were sent an invitation to participate in this study; 146 PERC participants across 15 chronic diseases completed the online survey (a 72% response rate). Self-reported participant demographics were diverse in terms of age, race/ethnicity, education level, and household income (Table 1). Demographic variables of age, gender, race, ethnicity, disease condition, and insurance types were assessed to ensure a diverse representation for focus group selection, and 36 respondents were invited to further participate in focus groups; 29 respondents agreed to participate. Characteristics of the focus group subset were similar to those of the survey respondents.

### 3.2. Health Insurance Coverage

The types of health insurance plans held by the participants are specified in Table 1. Most survey respondents (95/146 [65%]) and focus group participants (21/29 [72%]) had either employer-based or Medicare insurance only. The top three issues that influenced choice of insurance plan (for those who were able to choose) were choice of provider/hospital (85/133 [64%] participants), copay (49/133 [37%]), and quality of prescription medication coverage (44/133 [33%]).

### 3.3. Satisfaction with Coverage

Most survey respondents expressed satisfaction with their insurance: 118/146 (81%) were satisfied with their health plan in general, and 107/146 (73%) were satisfied with the clarity of information available to them about their coverage and costs of prescription medications. No discernable differences were observed in terms of satisfaction between respondents with Medicare and those with commercial insurance. Overall, 64/146 (44%) respondents agreed that their health insurance provider showed concern for their wellbeing and long-term health. Most respondents (111/146 [76%]) considered their health insurance coverage valuable/highly valuable given the cost of the premiums.

*“I think my insurance [Medicare plan] is good as it is, it could certainly use some major rewrites…As someone else said, when it works, it works well.”*

Psoriasis, female.

In the focus groups, participants shared more in-depth perspectives about the nature of their satisfaction changing over time and fluctuating with life events such as retirement, which would usually necessitate a change in insurance coverage. Financial wellbeing and the perceived value of insurance differed depending on employment status.

*“[Medicare catastrophic care] been very economical for what I pay in my premium that’s deducted from my retirement. The dollar-for-dollar value is by far much more than it was when I had the private insurance, when I was working for many years. Of course, my income working was a little higher because you’re working, but when you took out all the expenses, I’m actually in a better position financially now.”*

Psoriatic arthritis, male.

The demands of managing a chronic illness meant some participants needed to stop working and relied on their spouse’s income to cover insurance costs.

*“When I was first diagnosed with my disease, I had to retire from teaching…I had to do COBRA through my employer, only every month just for the premium was insane…thankfully my husband works and he provides financially for us to survive.”*

Pulmonary hypertension, female.

Approximately one-quarter of focus group participants expressed that their insurance provided little value, largely because it did not offer affordable access to medication. These participants shared how they had been impacted by specific gaps in coverage, including for mental health services. Concern was expressed about high OOP costs, even in what were perceived to be top-tier insurance offerings.

*“[The insurance] I picked with my employer is the top tier, like the very top insurance. So I’m paying a lot out of pocket out of each paycheck. But I feel like when I need the medication or go to get my prescription, it’s like a no win. I’m paying high cost for my insurance, but it’s still not necessarily helping me.”*

Inflammatory bowel disease, female.

### 3.4. Insurance Design

#### 3.4.1. Affordability and Access

Overall, 34/146 (23%) survey respondents said they struggled to afford the prescription medication covered by their health insurance plan, and of those who selected a reason for unaffordability, 33/93 (35%) identified cost sharing as a barrier and 22/93 (24%) identified high deductibles as a barrier. Responses showed no discernible difference in the effect of cost sharing on affordability between Medicare and commercial insurance. There was, however, a disproportionate effect of cost sharing based on annual income: half of all respondents with an annual income less than US$40,000 could not afford their OOP costs.

Problems associated with coverage were common. Within the past 12 months, 61/146 (42%) survey respondents had experienced a prior authorization, 42/146 (29%) had been denied medication coverage, 25/146 (17%) had experienced a non-medical switch, and 10/146 (7%) had undergone a step edit. Almost half (40/87 [46%]) of respondents attributed coverage barriers to financial rather than medical reasons.

**Figure 2:**
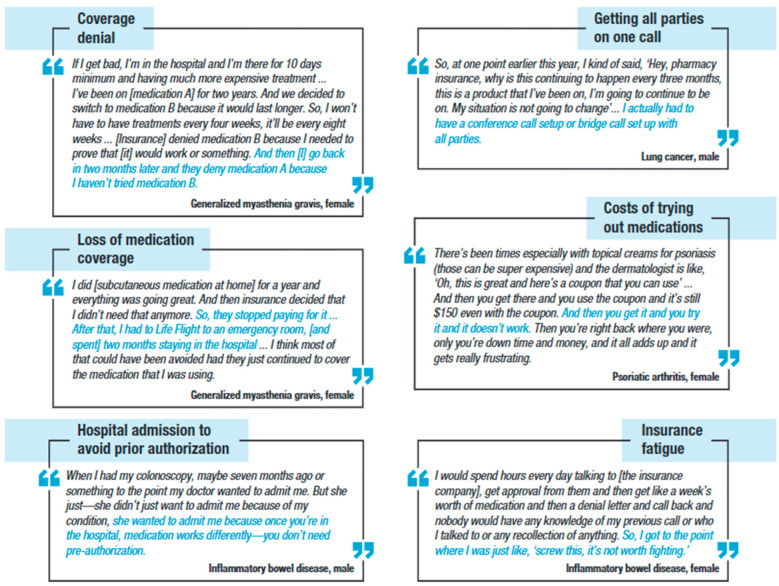
Illustrative focus group participant experiences with coverage and affordability scenarios.

Figure 2 presents illustrative quotes from focus group participants describing their experiences with insurance coverage and affordability. Some participants mentioned their frustration with prior authorizations. They felt it was wrong that the insurance companies, instead of their providers, made treatment decisions.

*“I want to rely on my doctor’s recommendation, which [is] the best one for me, not the cheapest that they can.”*

Venous thromboembolism, male.

Patients explained how denial of initial medication coverage often led to disease exacerbation and expressed frustration with paying premiums for insurance plans that could potentially deny them access to essential or life-saving medications. They shared personal experiences where they felt cost containment had conflicted with the appropriate provision of physician-directed care.

*“[My insurance] denied my medication. … And it’s just really scary. If I get bad, I’m in the hospital for 10 days minimum [taking] more expensive treatment. So, it’s just hard that they don’t take my doctor’s word for it. My doctor knows more about me than their doctors or whoever is making the decisions whether to cover it.”*

Generalized myasthenia gravis, female.

#### 3.4.2. Unpredictability of Cost

When discussing new medications with their provider, the vast majority (126/146 [86%]) of survey respondents remembered that clinical benefits were mentioned, but fewer than one-quarter (33/146 [23%]) recalled talking about cost. Overall, 23/146 (16%) respondents were not confident they understood how much they needed to pay for their prescription medication when selecting their health insurance plan. Despite 85/146 (58%) respondents researching costs before receiving their medications, 34/146 (23%) had never managed to find the information, and 30/146 (21%) were only able to source the information sometimes. In total, 84/146 (58%) respondents found that their actual medication costs differed from what they were expecting to pay.

Focus group discussions probed further into the challenges patients faced regarding medication costs. Participants cited difficulties associated with inconsistent/unclear pricing of prescription medication, changes in insurance formularies, and high OOP costs before deductibles. This was especially problematic for patients with rare diseases or those who needed a newly approved medication. One participant described issues with the accuracy of copay estimates.

*“I’ve never gotten an accurate estimate [for prescription cost] before. On their website, there’s a copay estimator tool, but neither of my medications are able to get an estimate because they’re uncommon, or the estimate has always been wrong.”*

Generalized myasthenia gravis, non-binary.

#### 3.4.3. Financial Burden

Of the 146 survey respondents, 16 (11%) reported spending more than US$2000 (not including premiums) over the previous 12 months on prescription medications, despite having insurance; five (3%) had spent more than US$5000. As a result, 27/146 (18%) survey respondents had reduced their spending for basic needs such as food and housing, an additional 19/146 (13%) had taken on debt to pay for medication in the previous 12 months, and 22/146 (15%) had abandoned a medication—OOP costs were the most common underlying reason. The financial strain of OOP costs on households disproportionately affected participants who were not White: 17/27 (63%) who reduced spending for basic needs, 15/19 (79%) who took on debt to pay for medication, and 15/22 (68%) who abandoned a medication were not White. The theme of financial burden was strongly echoed throughout the qualitative focus group discussions, with participants reporting negative impacts from cost exposure, as summarized in perspectives shared in Table 2.

#### 3.4.4. Emotional and Physical Impact of Financial Stress

**Figure 3:**
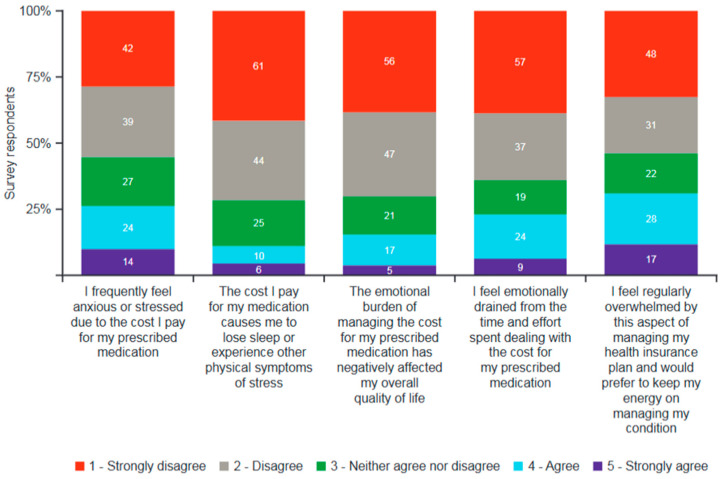
Emotional impact of managing cost of prescriptions over the previous 12 months (survey respondents; *n* = 146). Respondents replied using a scale of 1 to 5. Data labels within bars show number of patients.

One-quarter of survey respondents (38/146 [26%]) admitted feeling frequently anxious or stressed about the OOP costs associated with their prescribed medication, with some reporting loss of sleep, reduced overall quality of life, and feeling emotionally drained as a result (Figure 3). Nearly one-third (45/146 [31%]) felt overwhelmed by the cost of their health insurance plan.

**Figure 4:**
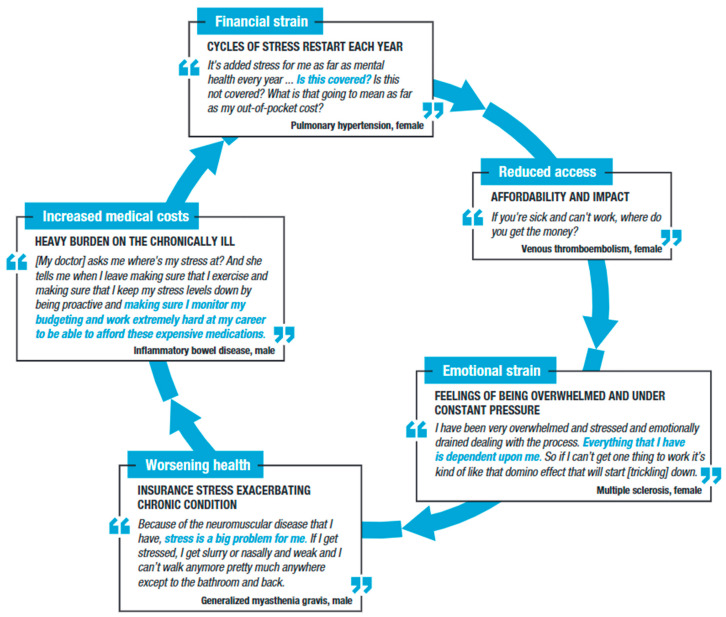
Stress/anxiety arising from inadequate insurance design throughout the patient journey.

The substantial emotional impact of managing prescription costs was evident in the focus groups. Participants described several challenges contributing to their stress and anxiety throughout the treatment journey, including uncertainty of coverage, failure to cover prescription medication costs, and difficulty troubleshooting issues with insurance companies. Collectively, these issues caused what participants referred to as overwhelming stress and anxiety, ultimately contributing to a sense of overall insurance fatigue (Figure 4).

*“My blood pressure went up, and I want to say this too. I had a light heart attack because of the stress and the worry and all that. It just really messed with me mentally. I got in a very depressed state.”*

Stem cell therapy, female.

Participants suggested that the stress caused by insurance issues was linked to increased disease activity or flares. Participants who skipped doses, stretched out medication, or abandoned medication altogether due to lack of access believed their health outcomes worsened as a result, and a common sentiment was that this diminished their chances of achieving optimal treatment outcomes.

*“I use [asthma medication] once a week when I’m supposed to use it daily because I just cannot cover that ‘cause it’s too much and I don’t have a copay card or anything to offset the cost. And so, what that leaves me with is constant worry that I’m going to have a breathing issue.”*

Venous thromboembolism, female.

### 3.5. Patient Perspectives on Potential Benefit of Design Reform

#### 3.5.1. Trade-Offs Are Acceptable to Reduce Cost Burden

Despite overall satisfaction with their coverage, most survey respondents agreed that revisions to insurance design were necessary, although they were pragmatic about the need to reduce the patient cost burden of medications and were open to trade-offs. A substantial majority (115/146 [79%]) indicated that they would prefer to reduce or eliminate any cost sharing, even if it meant slightly higher premiums for everyone. While it is difficult to contextualize these findings without a clearer explanation of what the term “slightly” might mean for individual patients, for every respondent preferring a lower premium, approximately four others wanted to slightly increase premiums to support a reduction in treatment OOP costs for patients. Most (119/146 [82%]) respondents stated they would be somewhat or very likely to pay more for their health insurance if it reduced their medication cost exposure; only nine (6%) respondents disagreed. The vast majority (140/146 [96%]) considered affordable access to new and innovative medications a somewhat or very important insurance benefit.

#### 3.5.2. Type of Cost Exposure Matters to Patients

Participants had mixed opinions about whether patients should bear any additional costs for medications if their doctor prescribed these based on recommended clinical guidelines. In total, 75/146 (51%) survey respondents considered a fixed copay to be an acceptable form of cost exposure. Only seven (5%) respondents considered it appropriate to have to pay (variable rates of) coinsurance. The remainder (64/146; 44%) believed they should not have to pay anything; however, this question failed to conceptualize any impact on premiums in this scenario. There was a clear preference for predictable and fixed costs in medication payment structures for patients with chronic disease: 76/146 (52%) respondents believed patients who were stable on a prescribed medication should not face any OOP costs for a refill, whereas 66/146 (45%) thought a fixed copay was reasonable. An overwhelming majority (142/146 [97%]) felt it was inappropriate to face (variable rates of) coinsurance at every refill.

When asked to consider potential consequences of eliminating patient OOP costs from current insurance designs, 114/146 (78%) respondents did not believe that patients would overconsume medications. In total, 114/146 (78%) agreed or strongly agreed that by removing OOP cost requirements, patients would be able to afford their prescription medication as prescribed by their doctor. When asked how their own behavior might change if they faced zero OOP costs for their prescribed medications, 130/146 (89%) respondents indicated they would just be focused on whether their medication works for them clinically.

#### 3.5.3. Multiple Policy Solutions Suggested

**Figure 5:**
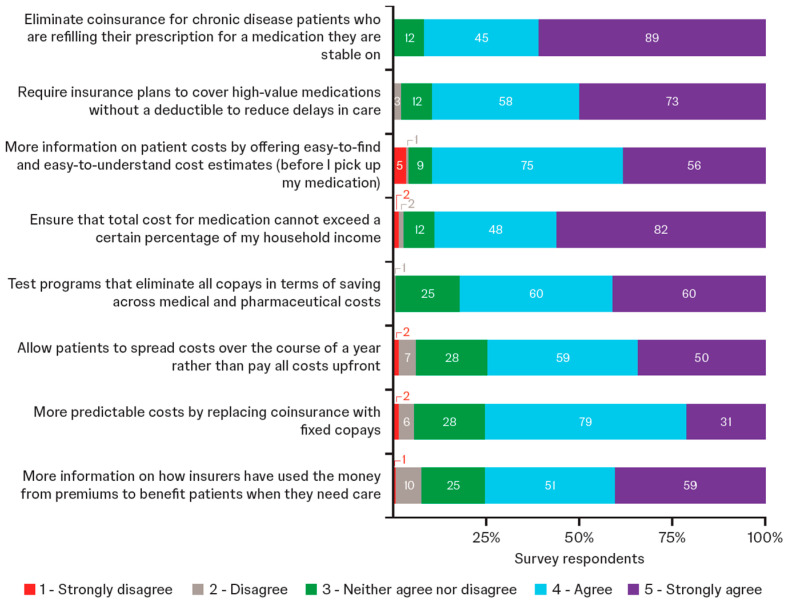
Level of support for proposed changes to health insurance (survey respondents; *n* = 146). Respondents rated the importance of each proposal using a scale of 1 to 5. Data labels within bars show number of patients.

Survey respondents broadly supported multiple policy solutions for redesigning health insurance to encompass reduced OOP costs, more transparency, and more user-friendly information (Figure 5). Levels of support for each of the potential solutions were similar among Medicare and commercially insured patients. Focus group discussions highlighted specific desires, certain misunderstandings, and the need for educational efforts to provide clear explanation of changes and how they might directly benefit patients. Participants acknowledged that the redesign of insurance may not lead to instant change (Table 3). Other solutions proposed by participants included universal, single-payer health insurance; personalized insurance that meets the needs of individual patients; removing the referral system (including elimination of prior authorizations) and the Medicare coverage gap; and allocating a point person to help navigate issues that can arise with insurance.

## 4. Discussion

Insights from patients with chronic disease around key insurance challenges and patient-centered solutions could help to inform health insurance design. This mixed-methods analysis captured broad perspectives from a group of patients with chronic disease via an online survey. Further exploration of individual context for additional granularity around specific topics took place within a smaller select group of patients via focus groups. Although most patients in this study were satisfied with their health insurance plan, they raised concerns about the unpredictability of costs. More generally, they had concerns about the affordability of prescription medication due to high deductibles and cost exposure, especially variable cost sharing and the disproportionate effects of OOP costs on those with lower income.

### 4.1. Perspectives on Insurance Design

Economic literature indicates that moral hazard-induced overconsumption is not a concern among patients undergoing chronic therapy, if their consumption is inelastic (i.e., if demand does not change in response to price). This study offers a patient-centered perspective on the rationale that requires patients with chronic disease to have “skin in the game” through cost-sharing mechanisms. A well-recognized challenge in financially empowering healthcare consumers is that in most clinical settings, patients are unable to determine OOP costs prior to receiving care [28]. In this study, participants clearly expressed that they often lacked the requisite information or negotiation power to be a driver of cost-effectiveness priorities in the healthcare system. Many participants in the focus groups explained that their OOP costs are unpredictable and essentially unknown at the time care is provided. Without such information, any type of cost exposure does not seem to empower patients but simply burdens those in greatest need. Study participants referred to existing payer management of their treatments as a tactic to prevent moral hazard, which they felt further diminished the likelihood of meaningful overuse.

Results of this study clearly indicated that participants were willing to accept trade-offs to address their cost burden. For those with chronic disease, an increase in premiums was preferable to leaving patients financially vulnerable and exposed in incomplete insurance designs. Quotes from the participants illustrate the consequences of indiscriminate cost-sharing mechanisms that placed a substantial financial burden on policyholders with chronic illness. Patient views in this study were consistent with previous research, which showed that cost sharing disproportionately impacted those with fewer financial means [29]. These perspectives offered vivid testimony that the perceived burden for patients with chronic disease can lead to unintended consequences, such as financial debt, therapy abandonment, poor treatment outcomes, and mental health issues.

This study found no discernible differences between patients with commercial insurance and those on Medicare; however, a disproportionate impact of cost exposure on patients with lower incomes was observed. Nearly half of survey respondents with an annual income of less than US$40,000 reported that they could not afford cost sharing for treatments, despite having insurance. This is consistent with previous work that showed that US citizens at the lower end of income distribution were most at risk financially from healthcare costs [2].

An important component of empowered healthcare decision-making is transparency about treatment costs. However, in this study, fewer than one-quarter of survey respondents reported ever discussing the cost of medication with their provider. This aligns with other literature that showed costs remained unaddressed in approximately two-thirds of patient–physician encounters [30,31,32,33]. Reasons for this may have included discomfort, fear of harming the patient–physician relationship or quality of care, lack of time, uncertainty about the physician’s role, and lack of knowledge about an individual’s financial and/or insurance circumstances [30,31,32,33]. Consequently, it is not surprising that two-thirds of survey respondents in this study revealed that the actual cost of their medication was not what they expected and, for some, led to abandonment at the pharmacy counter. Nearly half of the participants disclosed that they routinely arrived at the pharmacy unaware of the costs that they would face, despite their best efforts to find out beforehand. These findings have been documented in other studies, which have shown that patients were unable to ascertain costs until after the delivery of care [28,34], that a magnitude of therapy abandonment occurred at the pharmacy counter, and that there was a disproportionate burden on patients with lower incomes, especially among people who were not White [35,36].

It is important to remember that insurance cost-sharing models were introduced with dual objectives:

1. Keeping premiums low by shifting costs to people who use healthcare services; and
2. Discouraging excessive use of such services.

In this study, participants experienced high financial burden and reported a significant impact on their mental health and quality of life. One-quarter of participants described feelings of anxiety, and nearly one-third described being overwhelmed by the difficulties of managing the cost of their care. While roughly half of the participants accepted that fixed copays may be required as a mechanism to curb overuse and manage cost, participants considered variable cost sharing to present an inappropriate burden.

The present study raises the question of whether current benefit designs are compatible with the fundamental purpose of health insurance and its value (i.e., to reduce the financial impact of illness, thereby providing peace of mind to the insured). In this study, focus group participants were able to highlight the considerable financial hardship that patients with chronic disease faced when trying to meet OOP cost requirements, as well as the resulting anxiety that they experienced when managing their insurance product, routine costs, and access to therapy. Along with the results of the survey, these findings suggest that existing insurance plans may not be adequate to deliver on the promises of either a reduction in financial risk or access to treatment. Increased cost sharing has previously been associated with worse medication adherence and poorer healthcare outcomes in quantitative research [37,38]. The current study, which used in-depth patient insights, demonstrated that the challenges for these models were most pronounced for those with chronic disease, as these patients have frequent engagements with the healthcare system.

### 4.2. Perspectives on Reforms to Insurance Design

In the second part of this study, focus group participants discussed eight proposed reforms to insurance plans that were designed to cap or manage cost exposure and increase transparency of value:

1. Eliminate coinsurance for prescription refills;
2. Cover high-value medications without a deductible;
3. Fixed copays;
4. Limit medication cost to percentage of household income;
5. Test programs to eliminate copays;
6. Spread costs over a year;
7. More information on how insurers use premiums to benefit patients; and
8. Easy-to-find and easy-to-understand cost estimates.

Amongst these participants, there was strong support for multiple policy solutions to reduce treatment cost exposure for patients. Participants said they were looking for solutions that, instead of just restructuring how payments were made, reduced their overall financial burden. They indicated a need for policies that go beyond payment plan fixes. Participants were prepared to pay slightly higher premiums (i.e., have the entire insurance pool pay slightly higher premiums) for benefit designs that would be catered to their needs and others like them, which evoked discussion of fairness and equitable access to healthcare. Notably, for every survey respondent who preferred a reduction in premiums, there were four who preferred increased premiums to support lower medication costs. These qualitative insights align with recent quantitative policy research by employer organizations that suggests reducing cost sharing on high-value medications can have a relatively minimal impact on overall premiums [39,40].

This research offers an important differentiation on the type of cost exposure that matters to patients. In this study, participants were more receptive to fixed copays, with an overwhelming rejection of variable rates of coinsurance. Empirical research suggests that a reduction in OOP cost on needed medications may be valued broadly beyond those with the greatest health needs, estimating that US adults were willing to pay an extra $12.94 on average in insurance premiums per month for generous specialty-drug coverage [9]. Participants in this study also dismissed potential concerns that eliminating coinsurance from current insurance design may lead to inefficient consumption of medicines and increased overall costs (i.e., moral hazard). While we cannot quantify this sentiment in this work, a number of previous analyses lend empirical support to the notion that copay elimination for chronic disease medications does not necessarily increase total health spending, and may potentially decrease it, indicating there may be considerable offsets on total premiums due to a reduction in other healthcare costs [39,40,41,42,43]. While survey respondents supported increased transparency (i.e., “sunlight is the best disinfectant”), some focus group participants expressed skepticism that insurers would provide useful information for their healthcare decision-making, even if a policy mandated more detailed information on allocation of premium revenues or cost estimates.

### 4.3. Limitations

This research addresses the lived experiences of patients as they interact with health insurance. It did not seek to analyze underlying input drivers of insurance costs, which are, of course, important for policy reform and the subject of a rich body of literature involving quantitative economic and actuarial modeling. Understanding insurance terminology can be overwhelming, and there are inherent limitations when discussing complex and technical concepts with non-expert audiences. Considerable effort was made to explain certain terms within the survey questionnaire and focus groups to provide broader context and understanding. Any areas where ambiguity may have existed have been acknowledged transparently within the results and discussion sections. The results should be viewed as hypothesis-generating rather than hypothesis-confirming. Those invited to take part in this research were already participants in the sponsor’s PERC program and had volunteered to participate in this engagement. As such, there is potential for self-selection bias towards more disease-aware educated patients, who have greater familiarity with the insurance system, or those who may have had negative experiences in the past. Like all qualitative studies, this work is limited in its generalizability. Survey results may not be representative of the wider population of insured patients with chronic disease due to reliance on non-probability sampling, based on convenience; however, there was no indication that negative bias towards health insurance influenced the selection process, as evidenced also by participants’ expression of relatively high overall satisfaction with their plan.

## 5. Conclusions

Financial toxicity, defined as the patient-level impact of healthcare costs [44], can create a considerable barrier to successful management of chronic illness. Despite ongoing policy and academic discussions around insurance design and reform, there is a lack of patient-centered research and patient-driven debate. Because patients with chronic disease are disproportionately impacted, this exploratory research was conducted to document patient insights to inform and educate future discussions.

Although generally satisfied with the value of their health insurance, participants in this research (i.e., those with chronic disease covered by either commercial insurance or Medicare) explained the challenges that they faced due to their benefit design and patient cost exposure. Despite being insured, patients with chronic disease often lack access to medically necessary care, owing to deficiencies in insurance design and significant insurance barriers. As a consequence, they may be forced to incur medical debt or forgo essential care. The in-depth conversations presented in this study offer a more holistic, patient-centered perspective that contextualizes the statistical reports from broader survey research—findings that raise concerns about whether current benefit designs in the U.S. effectively leave many individuals functionally uninsured or underinsured [45]. In this study, participants perceived that the financial burden and obstacles that they faced related to insurance adversely affected their mental health and worsened their medical condition. These results align with previous concerns in which patients were described as “insured but not covered” [15,16].

Overall, survey respondents were pragmatic about potential reforms to insurance design, modest in their demands to make the burden of chronic disease less costly for them, and precise about their needs for greater predictability and transparency. These personal insights are valuable to guide novel approaches to improve current US health insurance designs. Participants endorsed several concrete approaches to address the issues they identified:

1. To eliminate coinsurance for individuals with chronic disease who need to refill prescription medications on which they are stable;
2. To require insurance plans to cover high-value medications without a deductible;
3. To expect insurance companies to provide more information on patient costs by offering easy-to-find and easy-to-understand cost estimates prior to medication collection; and
4. To ensure the total cost of medication does not exceed a certain percentage of a patient’s household income.

Unquestionably, these proposals for insurance redesign would require mathematical and actuarial analysis as well as trade-offs to balance the needs of those who rely on insurance the most and the broader ability of society to afford rising healthcare costs. However, this research offers valuable qualitative perspectives that should be incorporated in future decision-making about optimizing benefit design. This will be key to ensure patients’ needs are addressed in reforms for value-based insurance. While this research emerges from the intricacies of the US health insurance system, findings may resonate beyond the US as healthcare systems worldwide grapple with managing patient access and affordability questions when discussing reforms to benefit design and patient cost exposure.

## Figures and Tables

**Table 1:** Demographics and insurance type of survey respondents and focus group participants.

Parameter	Survey Respondents (*n* = 146)	Focus Group Participants (*n* = 29)
Age, years, median (range)	57.0 (23.0–81.0)	56.5 (31.0–80.0)
Age category, years, *n* (%)		
18–39	20 (14)	4 (14)
40–59	62 (42)	11 (38)
60–79	61 (42)	13 (45)
≥80	3 (2)	1 (3)
Gender, *n* (%)		
Female	91 (62)	17 (59)
Male	52 (36)	11 (38)
Non-binary	3 (2)	1 (3)
Race/ethnicity, ^a^ *n* (%)		
White	81 (55)	18 (62)
Black/African American	38 (26)	8 (28)
Hispanic/Latino	15 (10)	1 (3)
Asian American/Pacific Islander	7 (5)	2 (7)
Other	5 (3)	0
Highest level of education, *n* (%)		
Post-graduate	45 (31)	7 (24)
Bachelor’s degree	48 (33)	10 (34)
Associate degree	5 (3)	1 (3)
Trade school	9 (6)	2 (7)
Some college	28 (19)	6 (21)
High school	8 (5)	1 (3)
Other	3 (2)	2 (7)
Annual household income, ^b^ *n* (%)		
<$40,000	35 (24)	7 (24)
$40,000 to $79,999	27 (18)	7 (24)
$80,000 to $119,000	42 (29)	6 (21)
≥$120,000	34 (23)	8 (28)
Not sure/prefer not to say	8 (5)	1 (3)
PERC chronic disease category, *n* (%)		
Cardiovascular ^c^	24 (16)	5 (17)
Immunology ^d^	49 (34)	13 (45)
Neurology ^e^	15 (10)	1 (3)
Oncology ^f^	45 (31)	6 (21)
Pulmonary ^g^	13 (9)	4 (14)
Health insurance type, *n* (%)		
Employer-based only	56 (38)	12 (41)
Medicare only	39 (27)	9 (31)
≥1 insurance type	36 (25)	8 (28)
Medicaid only	11 (8)	0
TRICARE or Veterans Affairs only	2 (1)	0
Marketplace/Exchange only	2 (1)	0

^a^ Individuals could identify as more than one race/ethnicity. ^b^ In US dollars. ^c^ Cardiovascular: peripheral arterial disease, venous thromboembolism. ^d^ Immunology: ankylosing spondylitis, generalized myasthenia gravis, inflammatory bowel disease, psoriasis, psoriatic arthritis, stem cell therapy. ^e^ Neurology: major depressive disorder, multiple sclerosis. ^f^ Oncology: bladder cancer, lung cancer, multiple myeloma, prostate cancer. ^g^ Pulmonary: pulmonary hypertension. PERC, Patient Engagement Research Council.

**Table 2:** Participant quotes reflecting their experiences of the financial burden of out-of-pocket costs (focus group participants).

Patient Strategies to Afford Medication	Patient Perspectives
**Reduced household spending on food and bills**	*“I know how to be creative. I got to be creative with my bills [such as] food bills, because a big chunk of my check is going towards my medical, and I don’t want to be surprised by my medical bill when I’m being charged [so much].”* Psoriasis, male
	*“It could affect missing a bill, leave us short on grocery money, [force us to] do what we need to do until… we have the money.”* Inflammatory bowel disease, female
**Going without gas/electricity**	*“There was a couple of times my utilities were disconnected because I had to have this medication, and it was a hardship.”* Stem cell therapy, male
**Taking on debt**	*“I’m trying to figure out where I’m going to get money to get my kids Christmas presents. I don’t know how much room I have on my credit cards … it’s frustrating.”* Generalized myasthenia gravis, male
**Declaring bankruptcy**	*“I have filed bankruptcy twice in my life. I’m only 42. I mean, when you’re 18, and you’re $10,000 in debt, what do you do? … We spent over $10,000 [each year] on medication. And we did that three years in a row, and my husband only made $38,000 at that time. So, you can only imagine. We had no money.”* Inflammatory bowel disease, female
**Skipping doses or abandoning medication**	*“The insurance stopped covering it altogether, so that went way up… I had to stop that for quite some time until I could work that into the budget.”* Venous thromboembolism, female
	*“I’ll try to stretch [my meds] because I can only afford a month. I’ll just take half and try to get by until I can get the next month’s dosage.”* Inflammatory bowel disease, female
**Borrowing money**	*“I’ve encountered medications that were very difficult to afford and I had to borrow money from family to pay for them.”* Lung cancer, male
	*“I asked just somebody that I’m nearly a complete stranger with [for] $20 because I had to get to the pharmacy and get my medicine.”* Multiple sclerosis, female
**Patient assistance programs**	*“I was able to get a charity to help out with my medical costs now. So I have to pay it up front and then I can submit it for reimbursement and that helps so much.”* Generalized myasthenia gravis, male

**Table 3:** Patient perspectives on specific solutions for managing patient cost exposure (focus group participants).

Objective	Potential Solution	Discussion Summary	Patient Perspectives
**Cap cost exposure**	Eliminate coinsurance for chronic disease patients who are refilling their prescription for a medication they are stable on	Strongly supported by patients with chronic disease who appreciate the stability and predictability it would bring for medication costs Concerns: — Definition of “stable”—will insurers mandate patients remain on medication for a longer time (e.g., >1 year)? — Financial source for coverage—will premiums or other costs be raised? — May not benefit individuals with certain chronic conditions (e.g., experiencing flares, trying various medications, or using controlled substances that cannot be refilled)	*“The fixed copay, the[n] not hav[ing] to pay anything for a refill would be great because I’m on a lifetime of refill.”* Pulmonary hypertension, female *“The only thing I’m concerned about is the loophole, I guess that I see is …how long until they’re considered stable. [Insurers may mandate] that it would have to be a year or two years and then you’re just struggling.”* Pulmonary hypertension, female *“My medication, like for example, it costs like $150 or something like that out of pocket. And because it wasn’t necessarily working for me, when I went back to the doctor, my GI was like, well, let’s try this one. And this is within a week’s time. So here it is another medication that’s $150–200 something dollars, just because the first one didn’t necessarily work.”* Inflammatory bowel disease, female
	Require insurance plans to cover high-value medications without a deductible to reduce delays in care	Viewed favorably given the potential to prevent delays for patients who urgently need care but find deductibles a significant barrier Concerns: — Definition of “high value”—cost of medication or benefit to patient? — Financial source for coverage—will premiums or other costs be raised?	*“If [the price] goes outside of [the range of] what [insurers] think is okay, then they’ll question it or make it difficult for me to get it… Then I have to make decisions myself either asking for a lower dose or a generic version to get the cost down.”* Lung cancer, male *“Who decides what is my value? Would that be the patient? The doctor? The insurance company? I guess it would depend on who is deciding; if it’s the insurance company then they’re probably not going to put [benefit] on that list at all.”* Inflammatory bowel disease, female
	Replacing coinsurance with fixed copays	Widely favored for its predictability and ease of budget management due to predictable fixed copays Concerns: — Clarity needed around potential caps and exact copay amounts — Need for clear and comprehensive communication from insurers regarding changes so that patients fully understand financial obligations	*“I’ve stated my point about the whole fluctuations of copays and just knowing, I guess maybe it’s because the next 12 months brings a very budget minded approach to my life. I want to know what do I got to do for that budget.”* Ankylosing spondylitis, male
	Ensure that total cost for medication cannot exceed a certain percentage of my household income	Viewed by most participants as equitable, though some expressed concerns that the percentage cap might still be high for certain income brackets Some misunderstanding regarding the policy’s intent to lessen the burden on lower income households Concerns: — Definition of household income (i.e., individual or total?) — Definition of total costs (i.e., total healthcare costs?) — Need for clear and comprehensive communication, ensuring patients understand its aim to provide financial relief based on income levels	*“If I have $1200 a month to spend and I spend $700 on rent, and I spent $400 on food, that leaves me with a $100. So I’m not going to the movies and I’m not buying yoyos and stuff. You shouldn’t make me pay anymore [than what’s left].”* Generalized myasthenia gravis, male *“I just don’t never liked those, just because you make more money, you should have to pay more. I don’t know. Because a lot of things don’t take into account, other aspects of your life that you may need the money for different stuff like that.”* Generalized myasthenia gravis, female
**Management of cost exposure**	Test programs that eliminate all copays in terms of saving across medical and pharmaceutical costs	Participants appreciated the idea of eliminating cost exposure, but expressed concerns because copays contribute to deductible thresholds Some uncertainty was expressed due to confusion around the wording “test programs” Concerns: — Broader financial impacts and benefits — Financial source for coverage—will premiums or other costs be raised? — Need for clearer communication and education about potential advantages and trade-offs involved	*“I’m a little bit confused. What is meant by ‘test program’… I’m going to give a big thumbs down to them, to me it is more still trying to justify being [an] exorbitant cost.”* Venous thromboembolism, female
	Allow patients to spread costs over the course of a year rather than pay all costs upfront	Many participants were skeptical about a cost-smoothing approach, expressing concerns that this method subjects them to additional payment plans and does not address the underlying issue of total cost Concern: — Practicalities of medication switch	*“Every January everything starts over again. And my copays can get quite significant, like $500 a month, and if that’s the case, I cannot fill that prescription that month.”* Venous thromboembolism, male *“It’s great you could spread across the year, but if you’re spreading $20,000 across the year…it’s still an astronomical amount of money for families.”* Psoriatic arthritis, male *“I may be on a medication, but my body can reject it. And so now I need to be on another medication, but do I have three months’ worth of the other one that I need to pay? Or did I [already] pay for the portion of 12 months? So that could be kind of tricky there.”* Multiple sclerosis, female
**Transparency of value**	More information on how insurers have used the money from premiums to benefit patients when they need care	Participants acknowledged the intent behind increasing transparency around allocation of premium revenues, but remained skeptical about the practicality and reliability of this information and its utility in solving underlying issues of affordability Concern: — Need for more information and greater credibility/clarity around information provided by insurers	*“More information for patients on the use of their premium is needed, but it needs to come from the perspective of the patients as opposed to the insurer because coming from the insurer it can be more let’s say marketing and justification as opposed to transparency.”* Venous thromboembolism, female *“I just feel like unless there is some law or something that says a percentage of the premiums have to benefit patients in a certain way…does it really matter that they show us what they’re using it for?”* Psoriatic arthritis, female
	More information on patient costs by offering easy-to-find and easy-to-understand cost estimates	Some participants viewed the availability of clear and accessible cost estimates helpful to make informed decisions, particularly if available at the time their provider is prescribing a medication Overall enthusiasm was tempered by skepticism regarding the actual impact of this information on affordability Concerns: — Doubts about whether cost estimates would genuinely aid financial planning or decision-making — Numbers may be easy to fabricate/manipulate — Doubts about estimate accuracy for newly approved medications and impact of formulary changes	*“[I need to] see if it’s going to be covered, and what it will cost while I’m in the office with [my doctor]. And that helps me from getting to the pharmacy and saying, ‘Oh, I’m not picking that up’.“* Bladder cancer, female *“Doesn’t mean you can afford it…. The key word is estimates and they can change, and it varies by insurance companies and who they purchase from and more information on insurers.”* Psoriasis, male *“I think insurance companies are falling behind a little on the new medications and the coverages for them. I find it very difficult to get coverages of the new medicines. Like I said, that one they actually approved me for and then pulled it from the formulary.”* Psoriasis, male

GI, gastroenterologist; OOP, out of pocket.

## Data Availability

The data that support the findings of this research are available on request from the corresponding author. The data are not publicly available due to privacy or ethical restrictions.

## Supplementary Materials

The following supporting information can be downloaded at: https://www.mdpi.com/article/10.3390/jmahp13040056/s1: File S1: Online survey questions and answers; File S2: Discussion guide.

## Abbreviations

The following abbreviations are used in this manuscript:

AS	Ankylosing spondylitis
BlCa	Bladder cancer
gMG	Generalized myasthenia gravis
IBD	Inflammatory bowel disease
MDD	Major depressive disorder
MM	Multiple myeloma
MS	Multiple sclerosis
OOP	Out of pocket
PAD	Peripheral artery disease
PERC	Patient Engagement Research Council
PH	Pulmonary hypertension
PrCa	Prostate cancer
PsA	Psoriatic arthritis
PsO	Psoriasis
VTE	Venous thromboembolism
