# Supplementary material for: Patient Perspectives on Health Insurance Design: A Mixed-Methods Analysis
Source: J Mark Access Health Policy. 2025 Nov 14;13(4):56. doi: 10.3390/jmahp13040056 (PMC12641685; doi:10.3390/jmahp13040056)
Supplement: Supplementary file 1 [file jmahp-13-00056-s001.zip › jmahp-3885883-supplementary.pdf]

Journal of Market Access & Health Policy

MDPI

Supplementary Material

File S1. Online survey questions and answers.

Demographics

Please respond to the following questions about your health insurance coverage.

1. Please enter your full name. **[TEXT ENTRY]**

2. On a scale of 1-4, how familiar are you with the details about **your health insurance plan**? (such as health insurance coverage information, premiums and costs, and overall benefits)?

    () 1 – I know nothing **[TERMINATE]**
    () 2 – I know a little bit
    () 3 – I am somewhat familiar with the details
    () 4 – I am very familiar with the details

[PAGE BREAK]:

3. What was your annual household income for 2022?

    () Less than $20,000
    () $20,000-$39,999
    () $40,000-$59,999
    () $60,000-$79,999
    () $80,000-$99,999
    () $100,000-$119,000
    () $120,000 or more
    () I'm not sure/Prefer not to answer

4. Where do you get your health insurance plan? Please select ALL that apply. **[ALLOW MULTIPLE RESPONSES]**

    [] Through employer-based insurance
    [] Through a marketplace/exchange (e.g., self-employed)
    [] Through Medicare (**ADD TOOLTIP:** for people 65 and older or people with certain disabilities)
    [] Through Medicaid or any kind of government-assistance plan (**ADD TOOLTIP**: for those with low incomes or a disability)
    [] Through TRICARE or other military health care
    [] Through VA (enrolled for health care through the Veteran's Administration)
    [] I am uninsured
    [] Other (Please specify) **[OPEN FIELD]**

5. **[DISPLAY IF Q4 "MEDICARE, FOR PEOPLE 65 AND OLDER OR PEOPLE WITH CERTAIN**

DISABILITIES" SELECTED IN Q4] What type of Medicare plan(s) do you have? Select all that apply. **[ALLOW MULTIPLE RESPONSES]**

[] Traditional or Original Medicare (Parts A, B)
[] Medicare Part D (drug coverage)
[] Medicare Advantage Plan (sometimes called Part C)
[] Medicare Supplement insurance plan (i.e., Medigap)
() I'm not sure

6. **[DISPLAY IF Q4 "THROUGH EMPLOYER BASED INSURANCE" IS SELECTED]** How many different choices did your employer offer you during health plans enrolment?

() 1 **[SKIP TO Q8]**
() 2
() 3 or more
() I don't remember **[SKIP TO Q8]**

7. Please review the following list and choose the **top three issues** that influenced your decision in choosing your most recent health insurance plan. **[CHOOSE 3].**

[] Choice of providers and hospitals
[] Choice of treatment options
[] Copay amount **[TOOLTIP: COPAY]**
[] Quality of prescription medication coverage
[] Premium **[TOOLTIP: PREMIUM]**
[] Deductible **[TOOLTIP: DEDUCTIBLE]**
[] Range of benefits (i.e., mental health, alternative health)
[] The plan was easy to understand
[] The plan required minimal effort to sign up
[] Other **[TEXT ENTRY]**

8. Think back to when you selected your last health insurance plan. On a scale of 1-5 (where 1 is not at all satisfied and 5 is very satisfied), how satisfied were you with the clarity of information around the cost and coverage of prescription medication(s)?

1 – Not at all satisfied	2 – Not very satisfied	3 – Neutral	4 – Somewhat satisfied	5 – Very satisfied	Not sure/does not apply

9. Are other members of your family insured under your health insurance plan?

() Yes, other members of my family are insured under my health insurance coverage

() No, I am the sole member of my health insurance plan

Experience with Coverage

Tooltip definitions:

- "Health insurance premiums": the amount you pay for your health insurance plan every month, either directly or through your employer/ salary for your contribution for employer-provided coverage
- "Deductible": a specified amount of money that you must pay out of pocket before the health insurance plan starts paying
- "Copay": a fixed amount you pay for a service or prescription medication covered by your health insurance plan after you have paid your deductible, if applicable
- "Coinsurance": a percentage of the costs of a service or prescription medication you pay after you've paid your deductible, if applicable

Please respond to the following questions about your experience with your health insurance plan.

10. Does your health insurance plan include an annual deductible? **[TOOLTIP: DEDUCTIBLE]**
    () Yes
    () No
    () I'm not sure

11. **[DISPLAY IF "YES" SELECTED IN Q10]** Is that amount a family deductible or per person deductible? **[TOOLTIP: DEDUCTIBLE]**.
    () Family deductible
    () Per person deductible
    () I'm not sure

12. **[DISPLAY IF "FAMILY DEDUCTIBLE" SELECTED IN Q11]** What is your annual deductible? **[TOOLTIP: DEDUCTIBLE]**.
    () Less than $5,000
    () $5,000 to under $10,000
    () $10,000 to under $15,000
    () $15,000 to under $18,000
    () $18,000 or more

13. **[DISPLAY IF "PER PERSON DEDUCTIBLE" SELECTED IN Q11** What is your annual deductible per person? **[TOOLTIP: DEDUCTIBLE]**
    () Less than $2,000
    () $2,000 to under $3,000

() $3,000 to under $4,000
() $4,000 to under $5,000
() $5,000 to under $6,000
() $6,000 to under $7000
() $7,000 or more
() I'm not sure

14. When you pay for a prescription medication, which of the following do you pay:

() Copay [**ADD TOOLTIP: COPAY]** (a fixed amount you pay for prescription medication covered by your health insurance plan after you have paid your deductible, if applicable)
() Coinsurance [**ADD TOOLTIP: COINSURANCE]** (a percentage of the costs of a service or prescription medication you pay after you've paid your deductible, if applicable)
() Full price of prescription medication
() I'm not sure

15. Overall, how difficult is it for you to afford **prescription medication** covered by your health insurance plan?

1 – not difficult at all **[SKIP TO Q17]**	2 – not very difficult	3 – neutral	4 – somewhat difficult	5 – very difficult

(SKIP TO Q17 IF 1 is selected)

16. What reasons make it difficult to afford your **prescription medication**? Select all that apply. **[ALLOW MULTIPLE RESPONSES]**

[] My deductible is too high **[TOOLTIP: DEDUCTIBLE]**
[] I can't afford my coinsurance **[TOOLTIP: COINSURANCE]**)
[] I can't afford my copays **[TOOLTIP: COPAY]**
[] Have to pay the full cost of medication as my insurance doesn't cover my prescription medication
[] Other
[] None of these

17. Over the last 12 months, how satisfied were you with your health insurance plan?

1 – Not at all satisfied	2 – Not very satisfied	3 – Neutral	4 – Somewhat satisfied	5 – Very satisfied

18. "Please indicate your agreement with this statement: 'My health insurance provider shows concern for my personal wellbeing and long-term health.'

() "Strongly agree"
() "Agree"
() "Neither agree nor disagree"
() "Disagree"
() "Strongly disagree"

[PAGE BREAK]:

Access

19. Over the past 12 months, how much anxiety have you experienced over your ability to afford the medication you need?

1 – No anxiety at all	2 – Not much anxiety	3 – A little anxious	4 – Somewhat anxious	5 – Very anxious

20. Over the last 12 months, have you experienced any of the following from your health insurance plan? Please select all that apply. **[ALLOW MULTIPLE RESPONSES]**

[] Switched from one medication to another for reasons unrelated to the medication working for me (Non-medical switch) **[ADD TOOLTIP: NON-MEDICAL SWITCH]**
[] Forced to wait to fill a prescription medication performed until my request was reviewed and approved (Prior authorization) **[ADD TOOLTIP: PRIOR AUTHORIZATION]**
[] Required to begin with another medication before taking the medication my provider prescribed (Step Edits) **[ADD TOOLTIP: STEP EDITS]**
[] Told that the medication my provider prescribed was not covered (Exclusion Lists) **[ADD TOOLTIP: FORMULARY EXCLUSION LISTS]**
() No, I have not experienced any coverage issues **[SKIP TO Q22]**
() I'm not sure **[SKIP TO Q22]**

Tooltip definitions:

A. **Non-medical switching:** A patient who is stable on their prescribed medication will be changed to a different medication (not a generic of the same medication) for non-medical reasons. This means the switch is made for

reasons ***other than*** the original medication not working, side effects, or problems taking the medication as prescribed.

B. **Prior Authorization:** An insurance policy that requires review and approval of prescribed treatments before they are dispensed. Coverage may be denied upon review.

C. **Step Edits**: Requiring a patient to begin treatment with one medication, and "stepping up" to alternatives only when the first medication fails.

D. **Formulary Exclusion Lists:** When a health insurance plan excludes certain prescription medications from coverage.

21. If you experienced one of the situations in the previous question, what would you say was the reason behind the decision?

() A medical reason
() A financial reason
() No reason was ever given to me
() I'm not sure

[PAGE BREAK]:

Communications

22. Have you ever researched the cost of a medication before you went to a provider or tried to fill a prescription?

() Yes
() No

23. When you were most recently prescribed a new medication, which of the following did your provider discuss with you? Select all that apply. **[ALLOW MULTIPLE]**

[] Benefits of taking the medication
[] Cost of the medication
() None of the above

24. Have you ever asked a provider to prescribe you a specific medication?

() Yes
() No **[SKIP TO Q26]**

–

25. **(DISPLAY IF YES TO Q24)** Did the provider prescribe that medication for you?

() Yes
() No

26. Over the last 12 months, how often were the costs you paid for medication different from what you had expected when your provider prescribed it?

Never	Sometimes	Usually	Most times	Always	Does not apply

27. Over the last 12 months, how often were you able to learn how much you would have to pay for specific prescription medications before getting the medications?

Never	Sometimes	Usually	Most times	Always	Does not apply

28. How confident are you that when you choose a health insurance plan, you understand the cost you will pay for the prescription medications you may need?

() Not at all confident
() Slightly confident
() Moderately confident
() Very confident

29. Considering how much you pay for health insurance premiums **[TOOLTIP: PREMIUM]** each month, how would you rate the insurance coverage you receive?

() Highly Valuable: The coverage is more than worth the cost of my premiums.
() Valuable: The coverage is worth more than my premiums.
() Neutral: The value of the coverage is comparable or similar to my premiums.
() Less Valuable: The coverage is worth less than my premiums.
() Not at All Valuable: The coverage does not justify my premiums.

[PAGE BREAK]:

Financial Impact

30. Over the past 12 months, did you or someone in your household need to reduce household spending for basic needs (e.g. food, housing) to pay for **prescription medication**?

() Yes
() No

31. Over the past 12 months, did you or someone in your household need to take on debt to pay for **prescription medications**?

() Yes
() No

32. Over the past 12 months, did you or someone in your household have to abandon a prescription despite having health insurance for the following reasons? Select all that apply: **[ALLOW MULTIPLE] [TOOLTIP: CO-INSURANCE] [TOOLTIP: COPAY] [TOOLTIP: DEDUCTIBLE]**

[] Yes due to coinsurance
[] Yes due to copay
[] Yes due to not having hit my deductible at that point
() No

33. Over the past 12 months, approximately how much did your household have to pay for **prescription medication** (not including premiums **[TOOLTIP: PREMIUM]**) despite having a health insurance plan?

() We had no expenses
() Less than $100
() $100 to $499
() $500 to $999
() $1000 to $1999
() $2000 to $4999
() $5000 or more
() I'm not sure

34. Think of your experience managing the cost you pay for medication(s) over the last 12 months. Please indicate your level of agreement with the following statements:

	Strongly Disagree	Disagree	Neither agree nor disagree	Agree	Strongly agree
I frequently feel anxious or stressed due to the cost I pay for my prescribed medication.					
The cost I pay for my medication causes me to lose sleep or experience other physical symptoms of stress.					

The emotional burden of managing the cost for my prescribed medication has negatively affected my overall quality of life.					
I feel emotionally drained from the time and effort spent dealing with the cost for my prescribed medication.					
I feel regularly overwhelmed by the aspect of managing my health insurance plan and would prefer to keep my energy on managing my condition.					

Alternatives and Solutions

[NOTE TO PROGRAMMING: KEEP ENTIRE SECTION ON SAME PAGE]:

35. Please choose your preferred option to complete this statement. "If I have health insurance and my provider prescribes a medication that is recommended by appropriate clinical guidelines, then...."

() …I should have to pay coinsurance on the medication **[TOOLTIP: COINSURANCE]**
() …I should have to pay a fixed copay for the medication, but zero coinsurance **[TOOLTIP: COPAY] [TOOLTIP:COINSURANCE]**
() …I should not have to pay anything

36. Please choose your preferred option to complete this statement. "A chronic disease patient on health insurance who has been stable on a prescribed medication....."

() ... should have to keep paying coinsurance on the medication at every refill **[TOOLTIP:COINSURANCE]**
() ... should pay a fixed copay for the medication at every refill, but zero coinsurance **[TOOLTIP: COPAY] [TOOLTIP:COINSURANCE]**
() ... should not have to pay anything for a refill

[PAGE BREAK]:

37. Please indicate your level of agreement with the following statements:

	Strongly Disagree	Disagree	Neither agree nor disagree	Agree	Strongly Agree

If patients do not have to pay for coinsurance, they will take more medication than they need [TOOLTIP:COINSURANCE]					
If patients do not have to pay for coinsurance, they will be able to afford their prescription medication and take what their provider prescribed [TOOLTIP:COINSURANCE]					

[PAGE BREAK]:

Please review and answer each of the questions in each category related to insurance policy.

[TOOLTIPS FOR TABLE BELOW; NO NEED TO DISPLAY IN QUESTION]

Non-medical switching: A patient who is stable on their prescribed medication will be changed to a different medication (not a generic of the same medication) for non-medical reasons. This means the switch is made for reasons ***other than*** the original medication not working, side effects, or problems taking the medication as prescribed.

Prior Authorization: An insurance policy that requires review and approval of prescribed treatments before they are dispensed. Coverage may be denied upon review.

Step Edits: Requiring a patient to begin treatment with one medication, and "stepping up" to alternatives only when the first medication fails.

Formulary Exclusion Lists: When a health insurance plan excludes certain prescription medications from coverage.

38. On a scale of 1-5 (where is 1 not at all important and 5 is very important), how important do you believe the following solutions are related to issues with health insurance plans:

	1 – not at all important	2 – not very important	3 – neutral	4 – somewhat important	5 – very important
Eliminate prior authorization for situations where the medication is working as intended **[ADD TOOLTIP: PRIOR AUTHORIZATION]**					
Insurance must take patient's severity of disease into account					

when considering a prior authorization **[ADD TOOLTIP: PRIOR AUTHORIZATION]**					
Treat the healthcare provider's treatment recommendation as a higher priority than the insurance company's decision					

[PAGE BREAK]:

39. Please complete the sentence with your preferred answer: "In a world where my medication does not cost me anything…"

() I would be less focused on whether my prescribed medication is working for me
() I would not change my behavior – I would remain as focused on whether my medications are working for me as I currently am today
() I'm not sure

[PAGE BREAK]:

40. Review the following solutions. Thinking about changes that could be made to **prescription medication coverage**, choose the one solution that you agree with the **most:**

() Reduce or eliminate coinsurance and copay costs for those who require medications, everyone should pay slightly more in premiums **[TOOLTIP: PREMIUM] [TOOLTIP: COINSURANCE] [TOOLTIP: COPAY]**
() Slightly lower premiums for everyone, those who require medications should pay higher coinsurance and copay costs **[TOOLTIP: PREMIUM] [TOOLTIP: COINSURANCE] [TOOLTIP: COPAY]**

[PAGE BREAK]:

41. How likely would you be willing to pay **more** for your health insurance premiums **[TOOLTIP: PREMIUM]** if it meant having prescription medications covered with **fewer** access restrictions (such as prior authorization)?
    () Not at all likely
    () Not very likely
    () Somewhat likely
    () Very likely

42. How likely would you be willing to pay **more** for your health insurance premiums **[TOOLTIP: PREMIUM]** if it meant having to pay **lower or no cost** when you need medications?
    () Not at all likely
    () Not very likely
    () Somewhat likely
    () Very likely

Review the following statements and indicate whether you agree or disagree with each.
I would be willing to pay **more** for my health insurance premiums **[TOOLTIP: PREMIUM]** if:

	Strongly Disagree	Disagree	Neither agree nor disagree	Agree	Strongly Agree
... if it meant having prescription medications covered with **fewer** access restrictions (such as prior authorization)					
… if it meant having to pay **lower or no cost** when I need a prescribed medication					

[PAGE BREAK]:

43. Below are some proposed changes that health insurers could take. For each change, please indicate your level of support, thinking about what is most important to you and your family.

	Strongly Disagree	Disagree	Neither agree nor disagree	Agree	Strongly Agree
More predictable patient costs by replacing coinsurance with fixed copays **[TOOLTIP: COINSURANCE] [TOOLTIP: COPAY]**					
More information on patient costs by offering easy-to-find and easy-to-understand cost estimates (before I pick up my medication)					
More information on how insurers have used the money from premiums to benefit patients when they need care **[TOOLTIP: PREMIUM]**					
Ensure that total cost for medication cannot exceed a certain percentage of my household income.					

[PAGE BREAK]:

44. Below are some proposed changes that health insurers could take. For each change, please indicate your level of support, thinking about what is most important to you and your family.

	Strongly	Disagree	Neither	Agree	Strongly

	Disagree		agree nor disagree		Agree
Test programs that eliminate all copays in terms of saving across medical and pharmaceutical costs. **[TOOLTIP: COPAY]**					
Eliminate coinsurance for chronic disease patients who are refilling their prescription for a medication they are stable on **[TOOLTIP: COINSURANCE]**					
Require insurance plans to cover high value medications without a deductible to reduce delays in care **[TOOLTIP: DEDUCTIBLE]**					
Allow patients to spread costs over the course of a year rather than pay all costs upfront					

45. In terms of future healthcare, on a scale of 1-5 (where 1 is not at all important and 5 is very important), how important is access to new and innovative medications for you and your family?"

1 – not at all important	2 – not very important	3 – neutral	4 – somewhat important	5 – very important

File S2. Discussion guide.

janssen
Patient Engagement
Research Councils

POLICY
PATIENT ENGAGEMENT RESEARCH COUNCIL
(PERC) ENGAGEMENT #1:
PATIENT CENTERED INSURANCE DESIGN DISCUSSION

NOVEMBER XX, 2022

Confidential & Proprietary janssen 1

Agenda

- Welcome & Ground Rules (5 min)
- Introductions (10 min)
- Experience with Coverage (20 min)
- Financial Impact (20 min)
- Break (5 min)
- Alternatives and Solutions – Part 1 (20 min)
- Alternatives and Solutions – Part 2 (25 min)
- Alternatives and Solutions – Conclusion (15 mins)
- Closing (1 min)

janssen
Patient Engagement
Research Councils

Confidential & Proprietary janssen 2

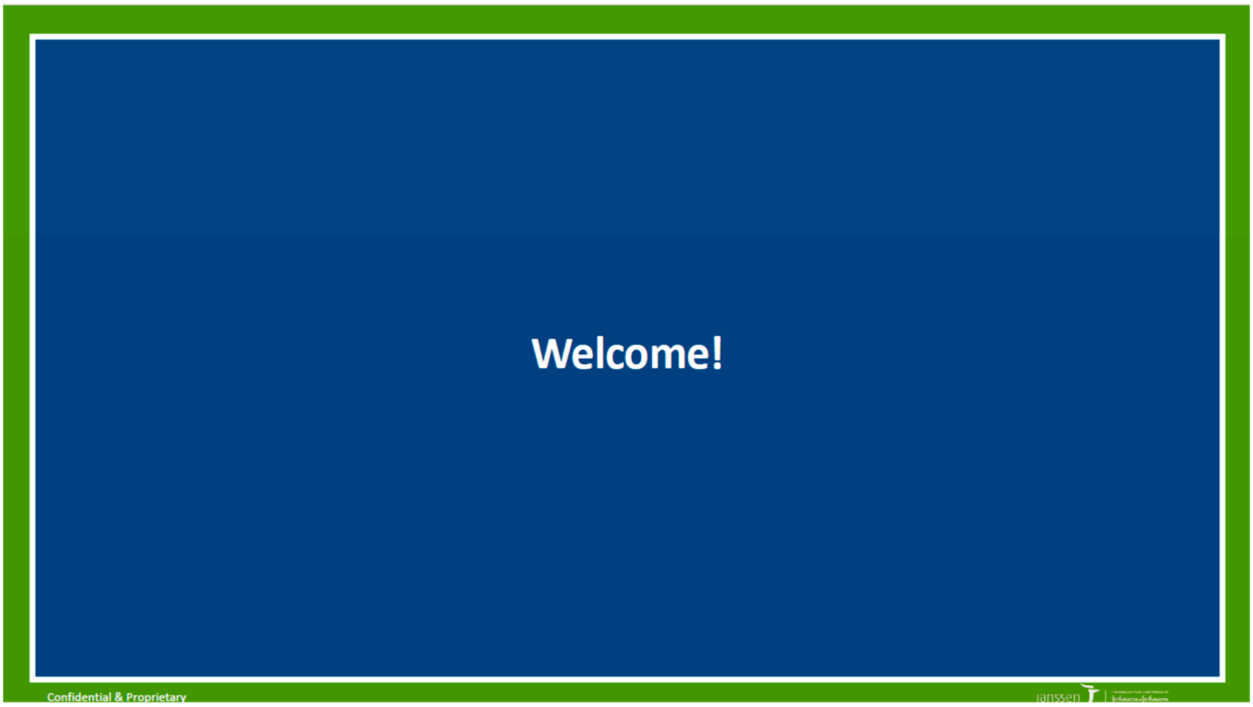
Welcome!
Confidential & Proprietary
janssen

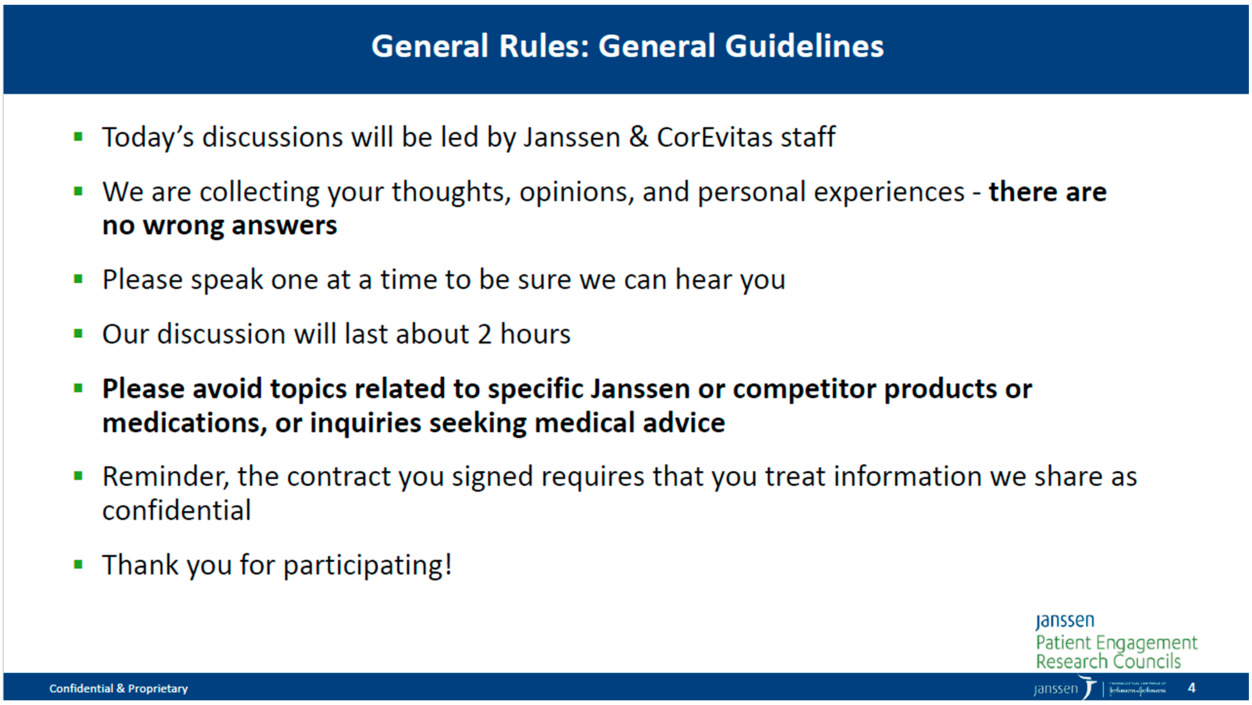
General Rules: General Guidelines
Today's discussions will be led by Janssen & CorEvitas staff
We are collecting your thoughts, opinions, and personal experiences - there are no wrong answers
Please speak one at a time to be sure we can hear you
Our discussion will last about 2 hours
Please avoid topics related to specific Janssen or competitor products or medications, or inquiries seeking medical advice
Reminder, the contract you signed requires that you treat information we share as confidential
Thank you for participating!
janssen
Patient Engagement
Research Councils
Confidential & Proprietary
janssen
4

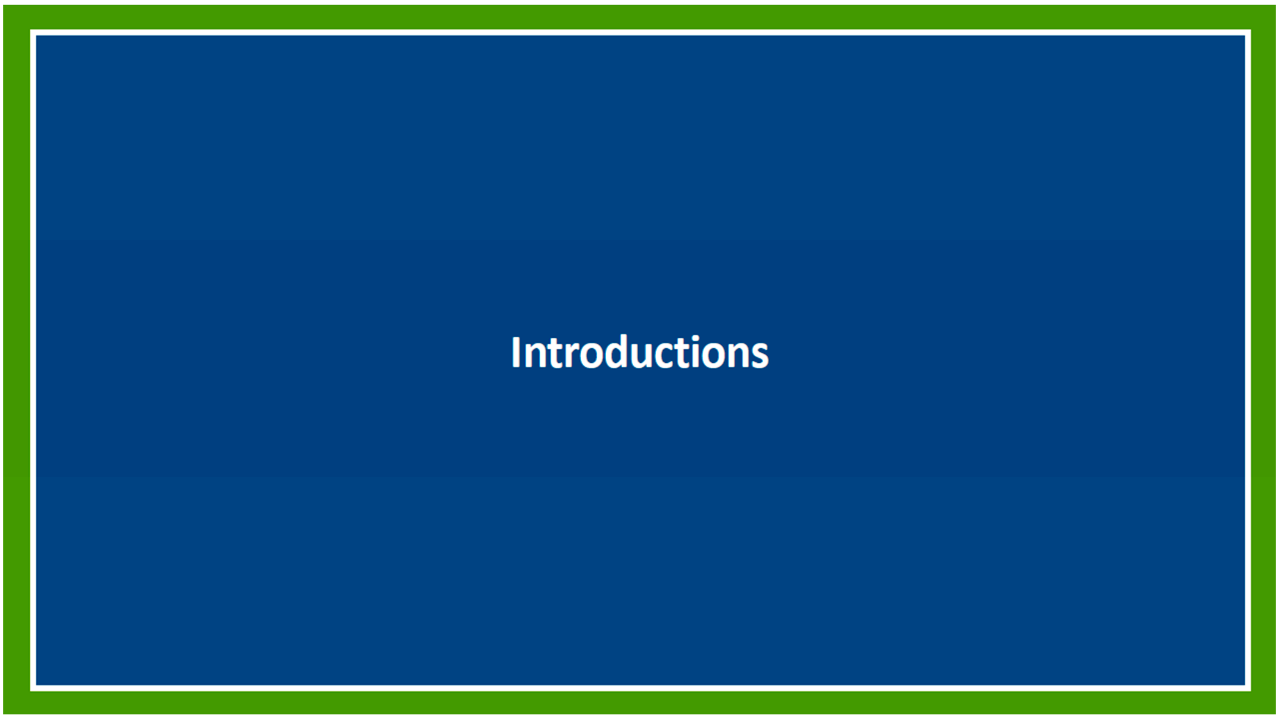
Introductions

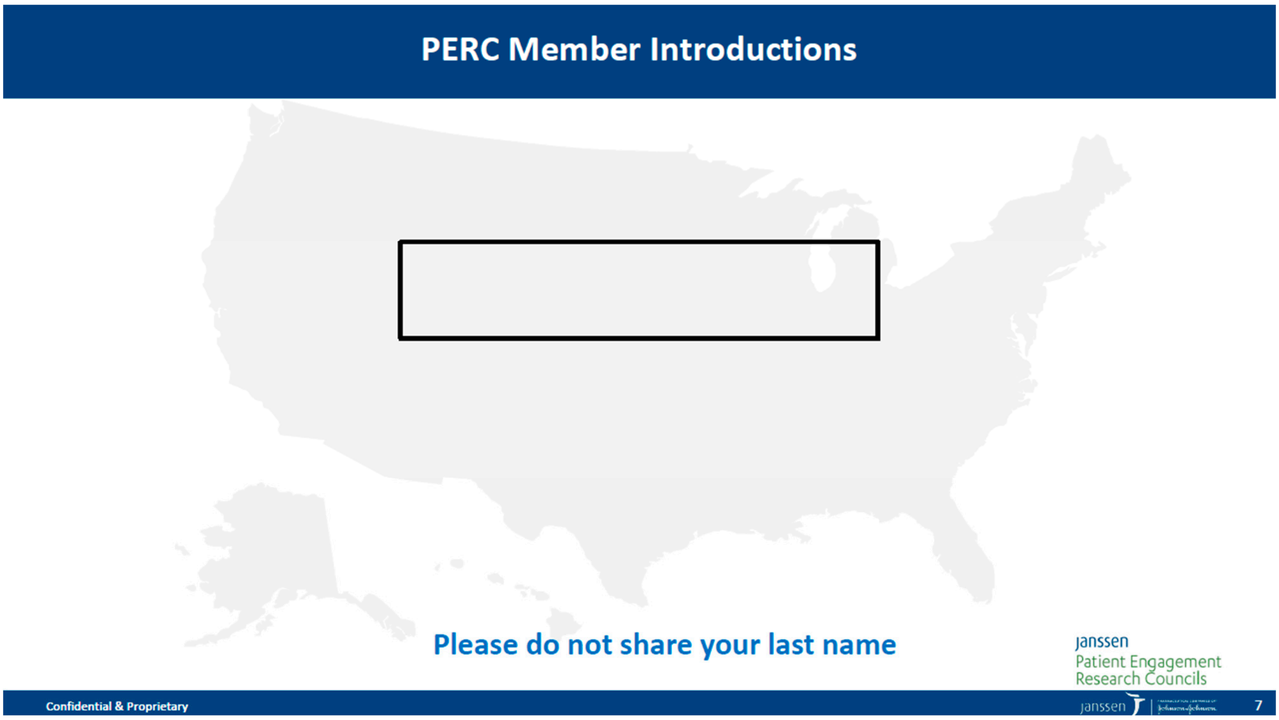
PERC Member Introductions
Please do not share your last name
janssen
Patient Engagement
Research Councils
Confidential & Proprietary
janssen
7

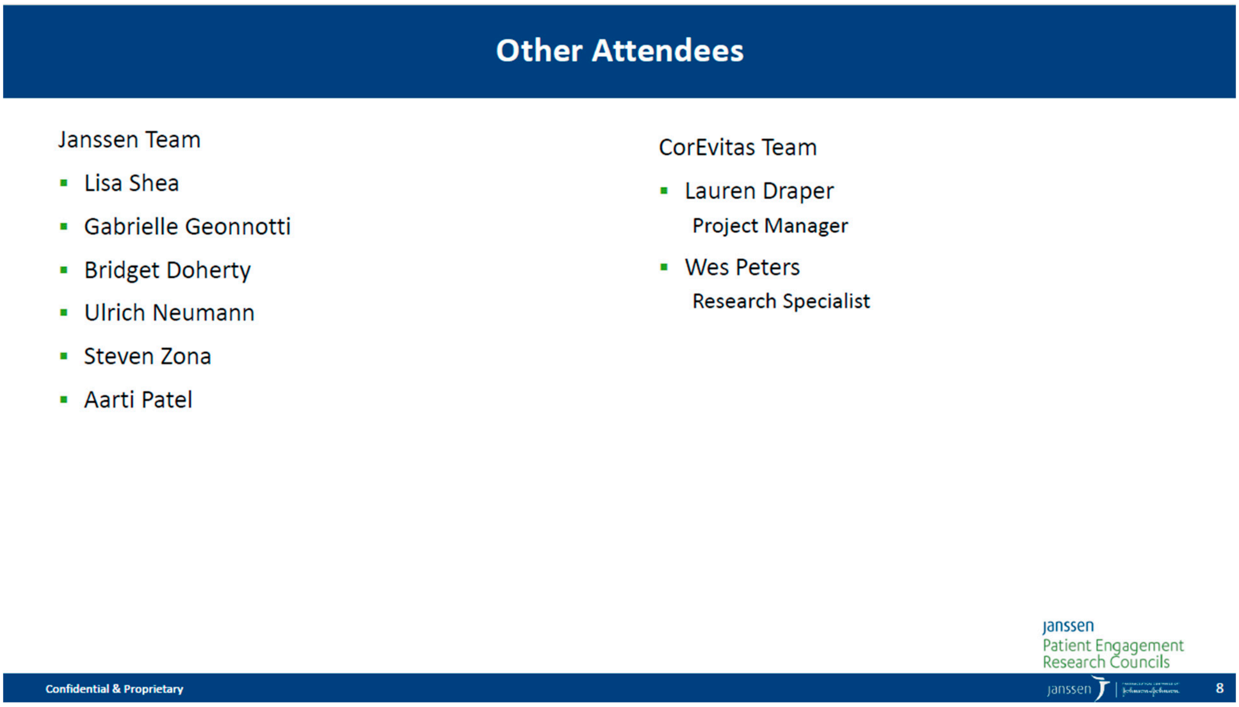
Other Attendees
Janssen Team
Lisa Shea
Gabrielle Geonnotti
Bridget Doherty
Ulrich Neumann
Steven Zona
Aarti Patel
CorEvitas Team
Lauren Draper
Project Manager
Wes Peters
Research Specialist
janssen
Patient Engagement
Research Councils
Confidential & Proprietary
janssen
8

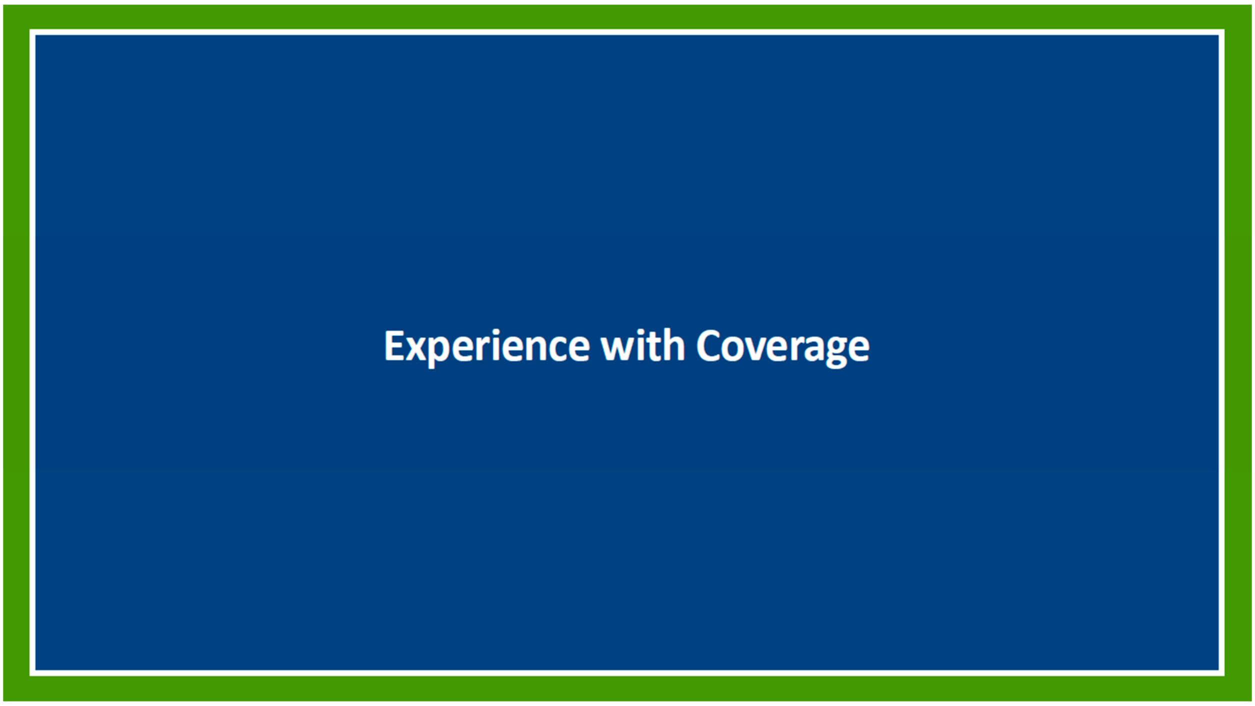
Experience with Coverage

Glossary of Terms

Deductible

- A specified amount of money that you must pay out of pocket before the health insurance plan starts paying

Copay/Coinsurance

- A fixed amount and/or percentage you pay for a service or prescription medication covered by your health insurance plan after you have paid your deductible.

Health Insurance Premium

- The amount you pay for your health insurance plan every month, either directly or through your employer/ salary for your contribution for employer-provided coverage

In-Network vs. Out-of-Network

- A provider network is a list of the doctors, other health care providers, and hospitals that a health insurance plan works with to provide medical care to its members.
  - These providers are called "network providers" or "in-network providers."
- A provider that isn't contracted with the plan is called an "out-of-network provider."

janssen Patient Engagement Research Councils

Confidential & Proprietary janssen 10

Experience with Coverage

Thank you for completing the Patient-Centered Insurance Design Survey!
Today we would like to share those findings with you and learn more about your thoughts on your insurance design.

For discussion:

- Costs for prescription medication
  - **24.2%** report "never" being able to learn how much they will have to pay for a prescription medication
  - **20.5%** report only "sometimes"
- Value of insurance coverage
  - **76%** rate their health insurance as "valuable" or "highly valuable" HOWEVER
  - **44%** agree their insurance provider shows concern for their wellbeing and long-term health
- Access Restrictions
  - **59.5%** experienced an access restriction (such as prior authorization, non-medical switch, denial, and/or step edits) in the past 12 months

janssen Patient Engagement Research Councils

Confidential & Proprietary janssen 11

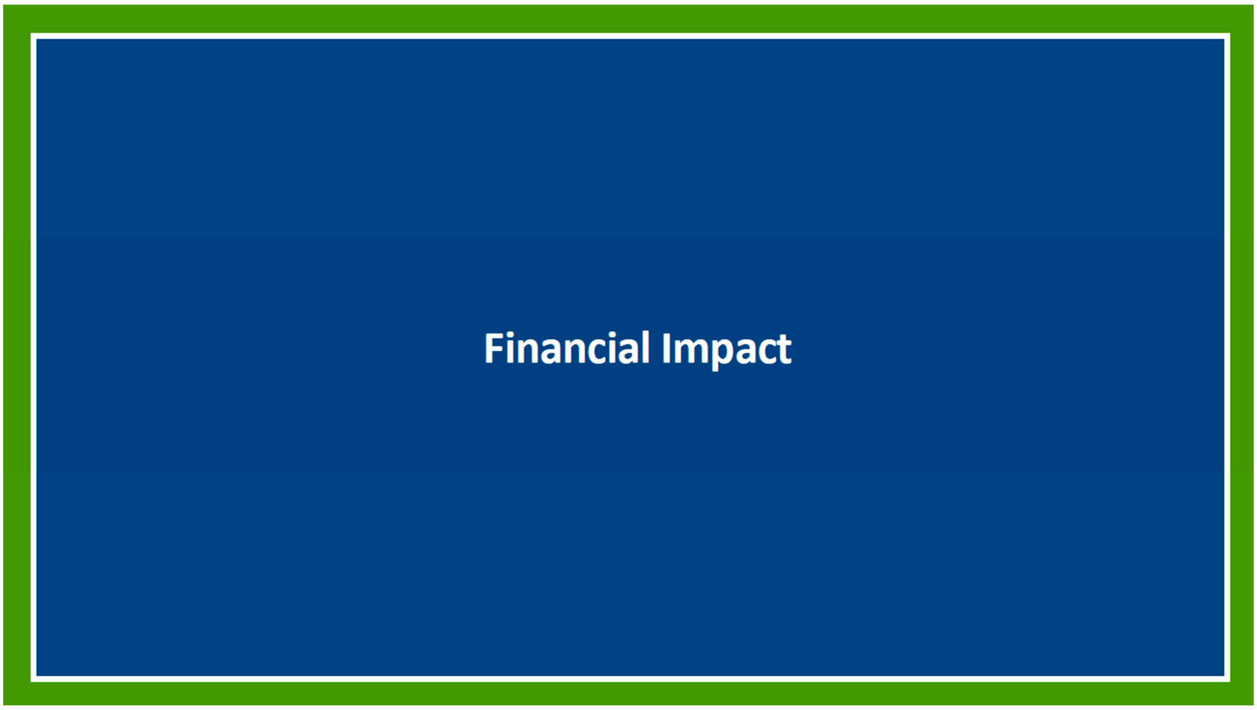
Financial Impact

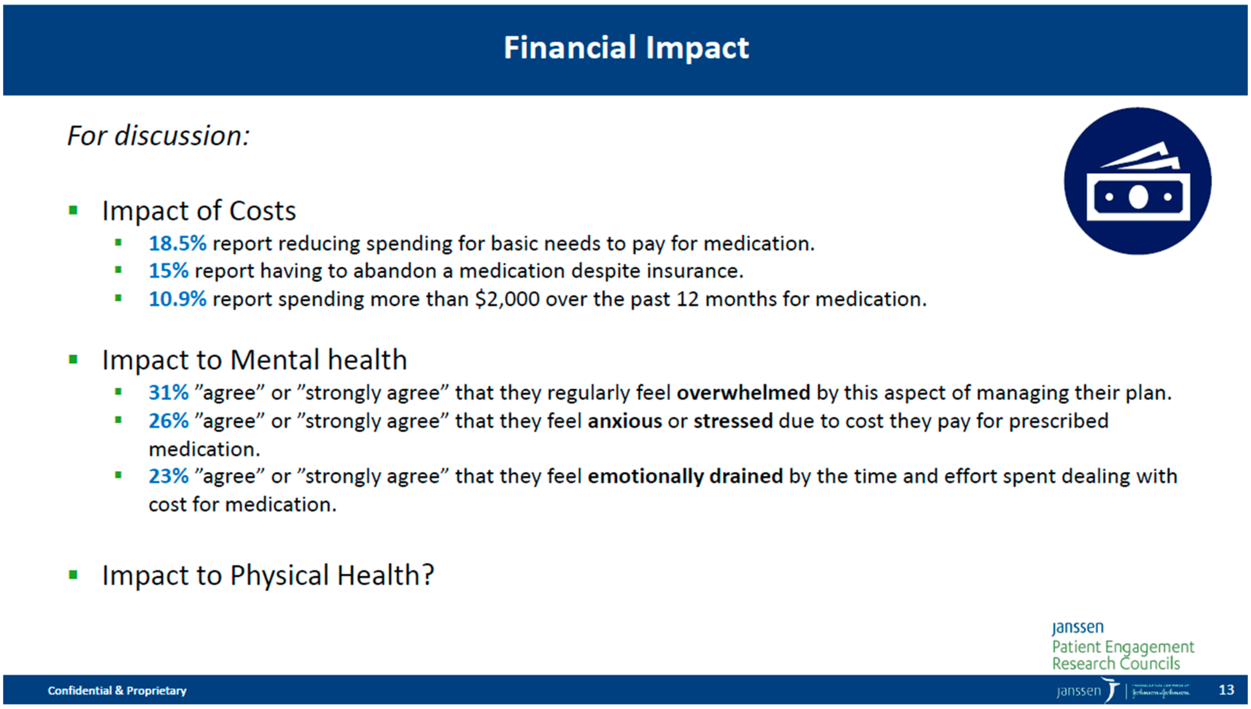
Financial Impact
For discussion:
Impact of Costs
18.5% report reducing spending for basic needs to pay for medication.
15% report having to abandon a medication despite insurance.
10.9% report spending more than $2,000 over the past 12 months for medication.
Impact to Mental health
31% ”agree” or ”strongly agree” that they regularly feel overwhelmed by this aspect of managing their plan.
26% ”agree” or ”strongly agree” that they feel anxious or stressed due to cost they pay for prescribed medication.
23% ”agree” or ”strongly agree” that they feel emotionally drained by the time and effort spent dealing with cost for medication.
Impact to Physical Health?
Janssen
Patient Engagement Research Councils
Confidential & Proprietary
janssen
13

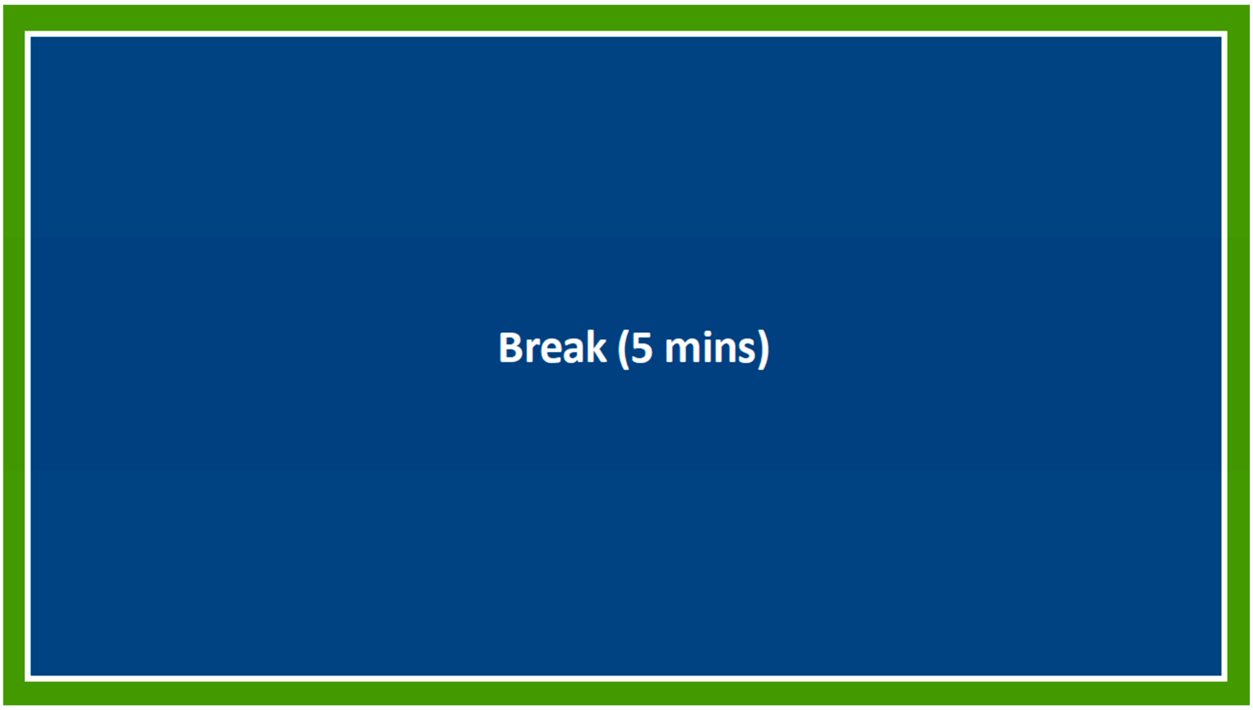
Break (5 mins)

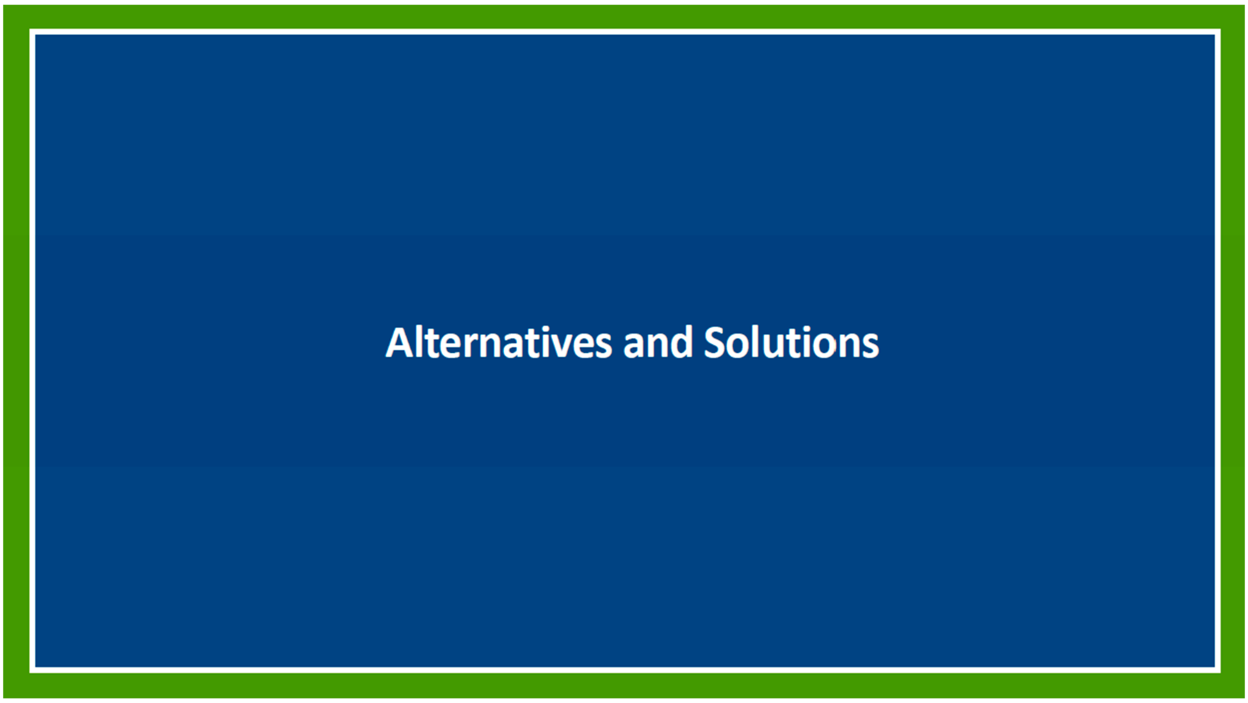
Alternatives and Solutions

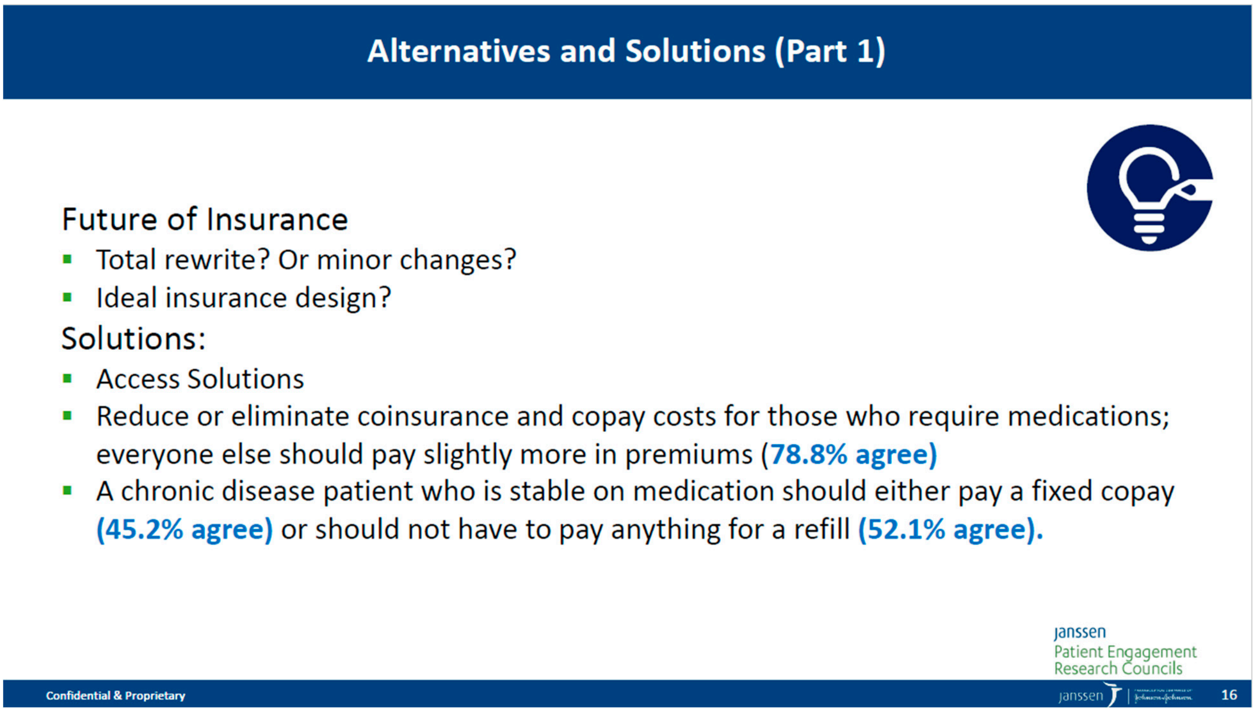
Alternatives and Solutions (Part 1)
Future of Insurance
Total rewrite? Or minor changes?
Ideal insurance design?
Solutions:
Access Solutions
Reduce or eliminate coinsurance and copay costs for those who require medications; everyone else should pay slightly more in premiums (78.8% agree)
A chronic disease patient who is stable on medication should either pay a fixed copay (45.2% agree) or should not have to pay anything for a refill (52.1% agree).
janssen Patient Engagement Research Councils
Confidential & Proprietary
janssen
16

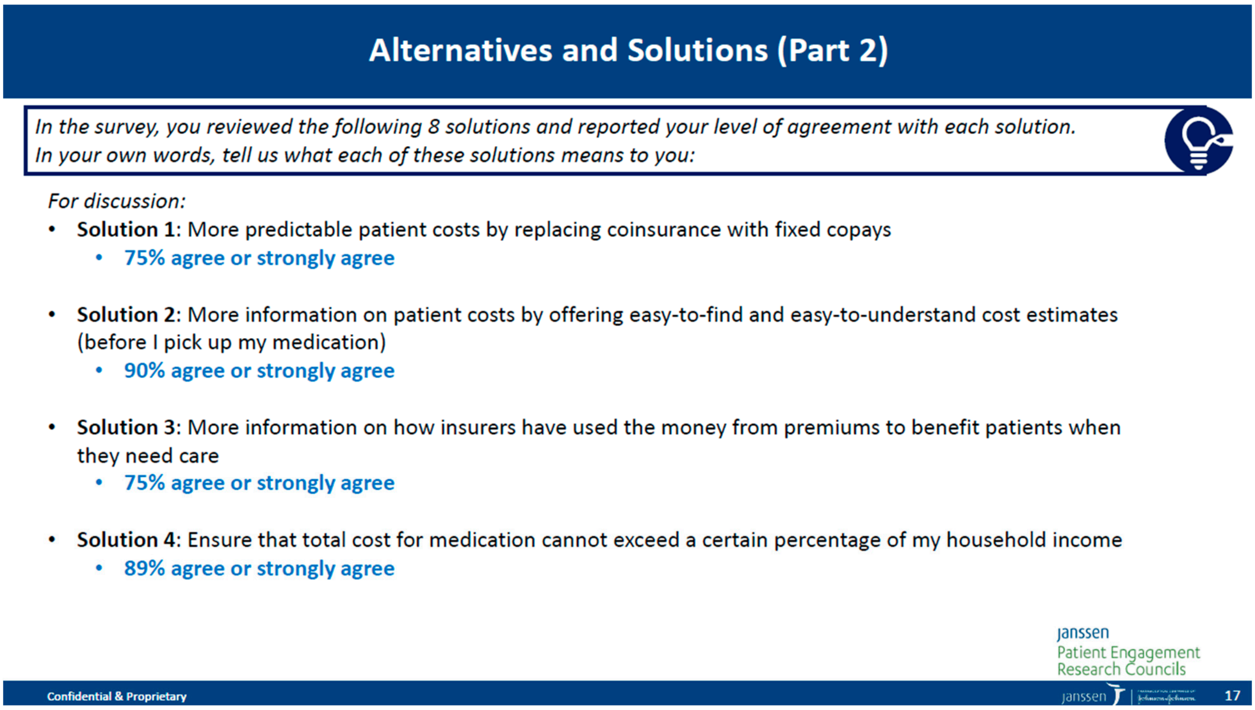
Alternatives and Solutions (Part 2)
In the survey, you reviewed the following 8 solutions and reported your level of agreement with each solution.
In your own words, tell us what each of these solutions means to you:
For discussion:
Solution 1: More predictable patient costs by replacing coinsurance with fixed copays
75% agree or strongly agree
Solution 2: More information on patient costs by offering easy-to-find and easy-to-understand cost estimates (before I pick up my medication)
90% agree or strongly agree
Solution 3: More information on how insurers have used the money from premiums to benefit patients when they need care
75% agree or strongly agree
Solution 4: Ensure that total cost for medication cannot exceed a certain percentage of my household income
89% agree or strongly agree
janssen Patient Engagement Research Councils
Confidential & Proprietary
janssen
17

Alternatives and Solutions (Part 2)

- **Solution 5**: Test programs that eliminate all copays in terms of saving across medical and pharmaceutical costs
  - **82% agree or strongly agree**
- **Solution 6**: Eliminate coinsurance for chronic disease patients who are refilling their prescription for a medication they are stable on
  - **92% agree or strongly agree**
- **Solution 7**: Require insurance plans to cover high-value medications without a deductible to reduce delays in care
  - **90% agree or strongly agree**
- **Solution 8**: Allow patients to spread costs over the course of a year rather than pay all costs upfront
  - **75% agree or strongly agree**

janssen
Patient Engageme...
Research Councils

Confidential & Proprietary 18

Alternatives and Solutions – Conclusion

- **Solution 1:** More predictable patient costs by replacing coinsurance with fixed copays
- **Solution 2:** More information on patient costs by offering easy-to-find and easy-to-understand cost estimates (before I pick up my medication)
- **Solution 3:** More information on how insurers have used the money from premiums to benefit patients when they need care
- **Solution 4:** Ensure that total cost for medication cannot exceed a certain percentage of my household income
- **Solution 5:** Test programs that eliminate all copays in terms of saving across medical and pharmaceutical costs
- **Solution 6:** Eliminate coinsurance for chronic disease patients who are refilling their prescription for a medication they are stable on
- **Solution 7:** Require insurance plans to cover high-value medications without a deductible to reduce delays in care
- **Solution 8:** Allow patients to spread costs over the course of a year rather than pay all costs upfront

Other ideas?

janssen
Patient Engageme...
Research Councils

Confidential & Proprietary 19

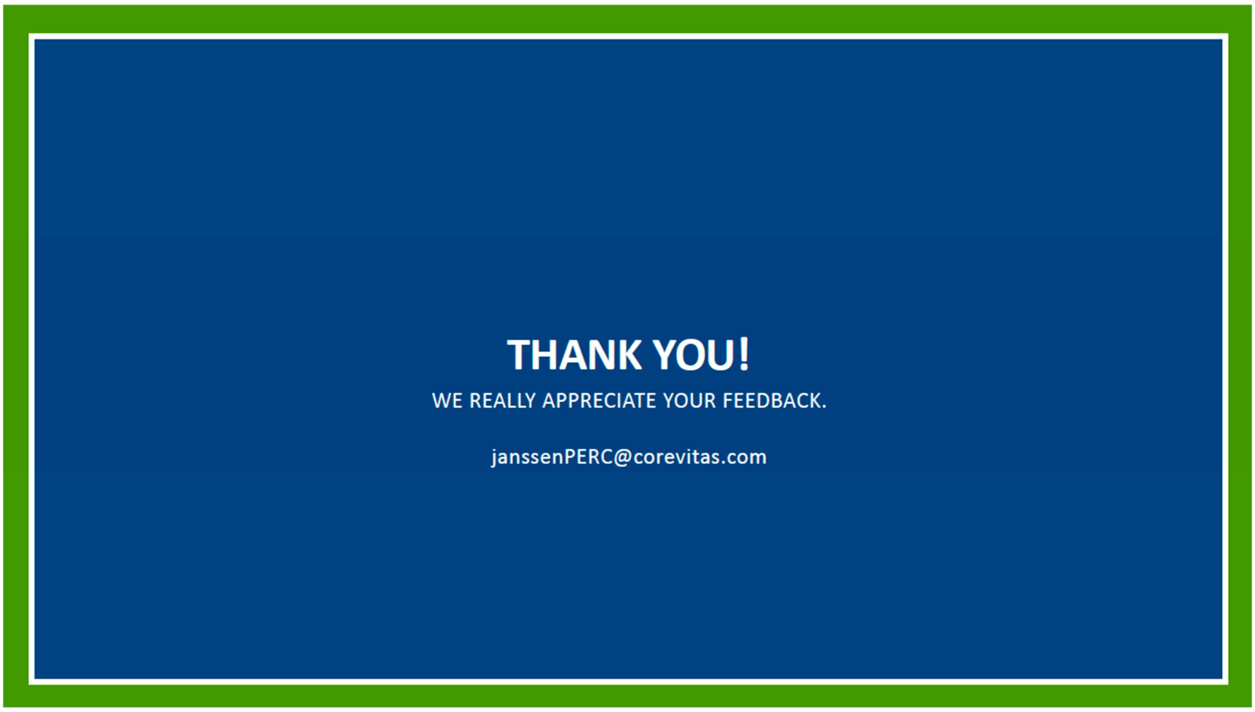

Disclaimer/Publisher's Note: The statements, opinions and data contained in all publications are solely those of the individual author(s) and contributor(s) and not of MDPI and/or the editor(s). MDPI and/or the editor(s) disclaim responsibility for any injury to people or property resulting from any ideas, methods, instructions or products referred to in the content.
